# Transcriptional activity of ammonia oxidisers in response to soil temperature, moisture and nitrogen amendment

**DOI:** 10.3389/fmicb.2024.1466991

**Published:** 2025-01-15

**Authors:** Chris Chisholm, Hong Di, Keith Cameron, Andriy Podolyan, Jupei Shen, Limei Zhang, Kosala Sirisena, Xueying Che

**Affiliations:** ^1^Centre for Soil and Environmental Research, Lincoln University, Christchurch, New Zealand; ^2^Key Laboratory of Humid Subtropical Eco-geographical Process of Ministry of Education, School of Geographical Sciences/School of Carbon Neutrality Future Technology, Fujian Normal University, Fuzhou, China; ^3^Research Center for Eco-Environmental Sciences, Chinese Academy of Sciences, Beijing, China

**Keywords:** Comammox *Nitrospira*, ammonia oxidizing bacteria, AOB, ammonia oxidizing archaea, AOA, nitrification

## Abstract

The contrasting response of AOA, AOB, and comammox *Nitrospira amoA* transcript abundance to temperature, moisture, and nitrogen was investigated using soil microcosms. The moisture, temperature, and nitrogen treatments were selected to represent conditions typically found in a New Zealand (NZ) dairy farm. AOB dominated all synthetic urine treated soils. Peak AOB *amoA* transcript abundance was positively correlated with estimated soil ammonia availability. While AOB gDNA abundance and nitrification rate trends were similar. AOA were strongly influenced by soil temperature. At 20°C, AOA *amoA* peak transcript abundance averaged over 1 order of magnitude higher than at 8°C. Within the AOA community a member of the *Nitrosocosmicus* clade was positively correlated with ammonium and estimated ammonia concentrations. The presence and relative increase of an AOA community member in a high nitrogen environment poses an interesting contrast to current scientific opinion in NZ. Comammox *Nitrospira* abundance showed no correlation with soil moisture. This suggests that previously found associations are more complex than originally thought. Further research is required to determine the drivers of comammox *Nitrospira* abundance in a high moisture environment. Overall, these results indicate that AOB are the main drivers of nitrification in New Zealand dairy farm soils.

## Introduction

Comammox *Nitrospira* are a newly discovered group of bacteria that are able to undertake both steps of the nitrification process, the oxidation of ammonia (NH_3_) via nitrite (${\text{NO}}_{2}^{-}$) to nitrate (${\text{NO}}_{3}^{-}$) (Van Kessel et al., 2015; Daims et al., 2015).

Originally, it was thought that comammox *Nitrospira* preferred substrate-limited conditions, such as ammonia-depleted biofilms (Van Kessel et al., 2015; Daims et al., 2015). However, subsequent studies have identified that certain members of the comammox *Nitrospira* community may favor a copiotrophic environment (Li et al., 2019; Chisholm et al., 2023). The environmental preference of comammox *Nitrospira* may be clade-specific, as several studies have suggested that comammox *Nitrospira* clade A and clade B respond differently to certain conditions, such as soil pH and ammonia availability (Wang et al., 2021; Li et al., 2022).

While some clade A members have demonstrated a positive response to nitrogen amendment (Orellana et al., 2018; Li et al., 2019; Takahashi et al., 2020), the response of clade B is less understood. Preliminary genomic studies suggest that clade B prefers a low nitrogen environment as their genome contains MEP-type ammonia transporters, which have a high affinity but low uptake capacity (Walter et al., 2008; Palomo et al., 2018; Li et al., 2022; Chisholm et al., 2024; Shah et al., 2024).

In terrestrial ecosystems, studies have shown that typically, clade A are more abundant than clade B (Li et al., 2022). However, Chisholm et al. (2023) found that comammox *Nitrospira* clade B was the most abundant ammonia oxidiser in a high-rainfall, high-fertility, pasture-based dairy farm soil and shared a strong positive correlation with soil moisture. This suggests that under certain environmental conditions, comammox *Nitrospira* clade B may be the dominant nitrifier.

There is a wealth of available information on ammonia oxidizing archaea (AOA) and ammonia oxidizing bacteria (AOB) in soil ecosystems (e.g. Prosser and Nicol, 2008; Di et al., 2009; Prosser and Nicol, 2012; Di et al., 2014; Taylor et al., 2017). While both are typically present in soil, they seemingly occupy different ecological niches. Historically, AOA are thought to prefer oligotrophic/extreme environments such as low ammonia availability, low pH, and high temperatures (Könneke et al., 2005; Francis et al., 2007; Di et al., 2009, 2010; Taylor et al., 2017). While AOB are functionally dominant in a nitrogen rich environment (Rütting et al., 2021). Within the context of New Zealand soils, AOB are typically more abundant than AOA (Di et al., 2009, 2010; Chisholm et al., 2023).

In order to investigate this further, an incubation experiment was conducted using a New Zealand dairy farm soil. The focus of the experiment was to compare comammox *Nitrospira* abundance, activity, and community composition to AOB and AOA under three different moisture levels: High (46.5% θg); Medium (37.5% θg); and Low (28.5% θg). Each moisture level was also run at two different temperatures to simulate the lowest (8°C) and highest (20°C) monthly average soil temperature in Canterbury, New Zealand. Two nitrogen levels were also employed to mimic common NZ dairy farm N inputs. These were urea-N fertilizer applied at 50 kg N ha^−1^ and synthetic urine-N, a concentrated localized application of N equivalent to 700 kg N ha^−1^ to simulate a dairy cow urine deposition during outdoor grazing (Cameron et al., 2013). Canonical ammonia oxidiser abundance, activity and active community composition was also analyzed to provide a useful comparison as the response of AOB and AOA to nitrogen amendment in various environments is well documented, and are generally contrasting (Di et al., 2009, 2010).

It is expected that comammox *Nitrospira* will share a positive and negative correlation with soil moisture and temperature, respectively. AOA will prefer the high temperature environment and AOB will dominate the synthetic urine treatments.

## Methods

A Templeton silt loam [typic immature pallic (Hewitt, 2010)] was used in this study. Soil from the top 10 cm was collected in May 2022 from the Lincoln University Research Dairy Farm (LURDF; 43°38′24.3″S 172°27′20.1″E; Lincoln, Canterbury New Zealand). The soil was sieved through a 6 mm sieve. A subsample was taken for soil physiochemical analysis (Supplementary Table 1).

### Physicochemical analysis of soil samples

Soil pH was determined by mixing soil and water at a ratio of 1:2.5. Samples were then shaken for 1 h and left to settle overnight. Soil pH was measured using a pH meter (Mettler-Toledo, Switzerland) the following morning. Olsen P was determined using the methodology explained in Olsen (1954). Soil cations (K, Ca, Mg, and Na) were quantified using a soil solution ratio of 1:20 1M ammonium acetate at pH 7 followed by atomic adsorption spectroscopy (Rayment and Higginson, 1992). Total N was analyzed by combustion method using an Elementor Vario Max Cube Analyser. The methods used to determine Organic C and CEC are explained in Blakemore (1987) and Brown (1943), respectively. Soil gravimetric water content was calculated by drying 10 g of soil at 105°C overnight. Soil ammonium, nitrate and nitrite were extracted using 2 M KCl. Briefly, 5 g of soil were mixed with 25 ml of 2 M KCl and shaken for 1 h. After centrifugation for 10 min, the supernatant was filtered through Whatman No. 41 filter paper. The solution was analyzed for ${\text{NH}}_{4}^{+}$ and ${\text{NO}}_{3}^{-}$ concentrations using a Flow Injection Analyser (FIA; FOSS FIA star 5,000 triple channel analyser). Soil nitrite concentration was not measured in this study. Ammonia availability was estimated using the Visual MinTeq modeling software (Gustafsson, 2011). The average nitrification rate was estimated by dividing the change in nitrate concentration by the number of days. The average nitrification rate was estimated by dividing the change in nitrate concentration by the number of days.

### Incubation experiment

Five-hundred grams (dry weight basis) was weighed into the plastic incubation containers (1 L volume, 10.0 cm diameter). Two holes were made in the lid of the containers to allow for gas exchange. The soil moisture was adjusted to 28.5% (L), 37.5% (M), and 46.5% (H) gravimetric water content [θ g; equivalent to 45%, 60%, and 75% water filled pore space (WFPS), respectively] and placed into an incubator set at either 8°C or 20°C to represent the Low and High temperature treatments, respectively. The soil was left in the incubators for 6 weeks for acclimatization before the nitrogen (N) treatments were applied. The following N-treatments were then applied: control, N0; 50 kg urea-N ha^−1^ equivalent, N50; and 700 kg synthetic urine-N ha^−1^, N700 (synthetic urine formula Clough et al., 1998). Each treatment had 4 replicates, totalling 72 experimental units. Samples were taken on days 1, 7, 14, 29, 57, 92, and 120. Soil pH, moisture, ammonia, nitrate, and AOB, AOA, and comammox *amoA* genomic DNA (gDNA) abundance were measured at each sampling date. AOB, AOA, and comammox *amoA* transcript abundance was measured from samples taken at days 7, 14, 29, and 57. Day 14 samples were selected for active AOB, AOA, and comammox community analysis. Soil samples used for molecular analysis were stored at −80°C for DNA and RNA extraction. The soil moisture content was maintained twice a week by monitoring the weight of the incubation vessels and adding deionised water to achieve the desired weight.

The genomic DNA (gDNA) was extracted from each sample using a NucleoSpin^®^ Soil Kit (Macherey-Nagel, Düren, Germany) following the manufacturer's instructions.

The extracted DNA was diluted 20-fold using deionised water in a CAS-1200 Robotic liquid handling system (Corbett Life Science, Australia), and stored at −80°C until ready for analysis.

The RNA extraction was conducted using RNeasy^®^ PowerSoil^®^ Total RNA Kit (QIAGEN GmbH, Hilden, Germany) according to the manufacturer's instructions. In short, 2 g of fresh soil was extracted using phenol/chloroform/isoamyl alcohol mixture (SigmaAldrich, St. Louis, MO, United States) and final RNA pellets were resuspended in 100 μl of elution buffer. The extracted total RNA was then treated with RNase-free DNase I (ThermoFisher Scientific, Waltham, MA, United States) according to the manufacturer's instructions. First-strand complementary DNA (cDNA) was synthesized using SuperScript IV One-Step RT-PCR I (InvitrogenTM, Thermo Fisher Scientific). The cDNA was diluted two-fold and stored at −80°C for downstream analysis.

Real-time quantitative PCR (qPCR) analysis was carried out using a QuantStudio™ 5 Real-Time PCR system (Thermo Fisher Scientific, Christchurch, New Zealand). Each qPCR reaction had a final volume of 16 μl containing 8 μl PowerTrack SYBR Green Master Mix (Thermo Fisher Scientific, Christchurch, New Zealand), 3.2 μl Invitrogen UltraPure Distilled H_2_O (Life Technologies, New York USA), 0.65 μl of each primer and 3.5 μl gDNA or cDNA template. Standard curves were generated using 10-fold serial dilutions of plasmids containing correct inserts of the target genes. Controls (DNase treated RNA) were included to ensure all DNA was removed from the RNA. The primer sets comamoA F/comamoA R (Zhao et al., 2019), Arch—amoA F/Arch—amoA R (Francis et al., 2005), and amoA1F/amoA2R (Rotthauwe et al., 1997) were used to quantify comammox *Nitrospira*, AOA, and AOB *amoA* gene abundances, respectively. The amplification conditions are given in Supplementary Table 2. Melting curve analysis was performed at the end of each amplification cycle to confirm the reactions specificity. Real-time qPCR data analysis was carried out by using QuantStudio Design and Analyse Software (Thermo Fisher Scientific, Christchurch, New Zealand).

Day 14 cDNA was selected for Next Generation Sequencing (NGS). Before sequencing, the samples underwent two rounds of PCR. The first round used adapted ComamoA F/ComamoA R, Arch-amoA F/Arch-amoA R, and amoA 1F/amoA 2R—overhang primers for comammox, AOA and AOB, respectively. The second used Nextera XT Index Kit (Illumina, San Diego, CA, USA) to attach barcodes to the sequences from each sample. The resulting PCR products were purified using AMPure XP beads (Beckman Coulter, Brea, CA). MiSeq Reagent kit v3 was used for library construction. The libraries were sequenced via the MiSeq platform (Illumina, San Diego, CA, USA). Sequencing was undertaken by Auckland Genome Services (Auckland, New Zealand).

The sequencing reads were imported into QIIME2 (version 2021-11) (Bolyen et al., 2019). Low-quality sequences with a quality score < 20, ambiguous nucleotides, short and chimeric sequences were discarded using the DaDa2 pipeline (Callahan et al., 2016). Amplicon Sequence Variants (ASVs) with < 0.005% relative abundance were removed (Bokulich et al., 2013). The ASVs were exported into the Geneious Prime software (version 2022.0.2), where the nucleotide sequences were *in silico* translated into amino acid sequences. Protein ASV sequences along with reference protein sequences collected from the NCBI database were used to construct a Bayesian phylogenetic tree (Supplementary Figures 3–5) (Huelsenbeck and Ronquist, 2001). Produced ASVs are given in the Supplementary material.

### Statistical analysis

Prior to statistical analysis, the transcript and gDNA *amoA* abundance values were log_10_ transformed. Repeated measures analysis of variance (ANOVA) were used to investigate the effect of temperature, moisture, and nitrogen on comammox *Nitrospira*, AOA and AOB transcript and gDNA *amoA* gene abundance over time.

A two-way ANOVA followed by an LSD test was used to determine if temperature, moisture, or nitrogen significantly affected the transcript and gDNA abundance of comammox, AOA, or AOB *amoA* at each sampling date (Genstat 22nd edition) (VSN International, 2022).

The Shannon alpha diversity indices were calculated in R with the “Vegan” package to summarize the diversity of active AOB, AOA, and comammox within samples (Dixon, 2003).

Weighted unifrac and Bray-Curtis analysis were conducted using the Qiime2 “qiime diversity core-metrics-phylogenetic” plugin (Bolyen et al., 2019). Heatmaps were produced in Qiime2 using the “qiime feature-table heatmap” plugin (Hunter, 2007). For AOA and AOB, two heatmaps were produced. One using the common (present in all experimental units) and the other using the clades obtained from the phylogenetic tree. Relative abundance was log_10_ transformed.

Using the “Hmisc” package in R, the Spearman correlation was employed to investigate any correlations between ASV relative abundance, environmental parameters, and the various nitrogen species concentrations (Harrell Jr and Harrell Jr, 2019). Only ASVs that were present in more than one-third of the samples and had more than 0.1% relative abundance were included.

The Spearman correlation was also employed to investigate the correlations between Shannons entropy and environmental parameters.

## Results

### *amoA* gene transcript abundance

#### AOB

**Figure 1:**
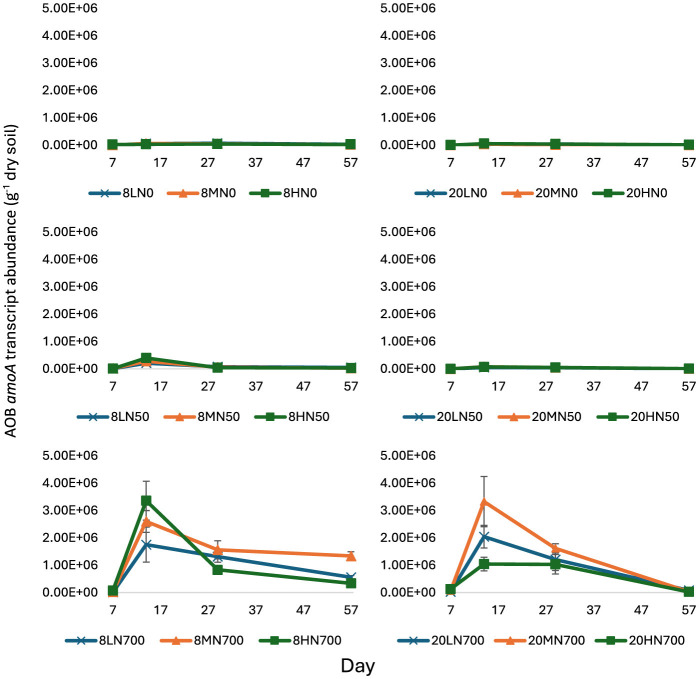
AOB *amoA* transcript abundance from day 7 to 57. Error bars represent standard error of the mean (*n* = 4). 8, 8°C; 20, 20°C; L, low moisture (28.5% θ g); M, medium moisture (37.5% θ g); H, high moisture (46.5% θ g); N0, no added nitrogen; N50, N applied at a rate of 50 kg N ha^−1^ as urea; N700, N applied at a rate of 700 kg N ha^−1^ as synthetic urine.

Throughout the trial, AOB *amoA* transcript abundance was significantly influenced by nitrogen (*P* < 0.001, v.r. = 149.79), while temperature (*P* = 0.029, v.r. = 5.56) and moisture (*P* = 0.012, v.r. = 3.27) had a relatively small effect (Figure 1). While AOB *amoA* transcript abundance significantly increased in response to N700, there was no significant response in the N0 and N50 treatments (Figure 1).

At day 7, moisture, nitrogen, and temperature did not significantly affect AOB *amoA* transcript abundance.

At day 14, AOB *amoA* transcript counts peaked at 3.36 × 10^6^ g^−1^ dry soil (8HN700) and 3.33 × 10^6^ g^−1^ dry soil (20MN700) for the 8 and 20°C treatments, respectively (Figure 1). Peak AOB *amoA* transcript abundance was one order of magnitude higher in the N700 treatments, relative to their respective N0 treatments. AOB *amoA* transcript abundance decreased after day 14.

At day 57, temperature (*P* < 0.001, v.r. = 146.64), moisture (*P* < 0.001, v.r. 22.35), and nitrogen (*P* < 0.001, v.r. = 149.51) had a significant effect on AOB *amoA* transcript abundance (Figure 1).

At all tested time points, AOB *amoA* transcript abundance exhibited a positive correlation with estimated ammonia concentration (expressed as mg L^−1^), amount of ammonium (expressed as kg N ha^−1^), and soil pH (except for day 29; Table 1). At day 14, the correlation between AOB *amoA* transcript abundance and soil NH_4_ and estimated NH_3_ concentrations was 0.85 and 0.74, respectively.

**Table 1:** Spearman correlation matrix for AOB, AOA, and Comammox *Nitrospira* (COM) *amoA* transcript abundance and select environmental parameters from day 7 to 57.

**AOB**				
	7	14	29	57
pH	0.58	0.53	–	0.50
NH_3_-N	0.60	0.74	0.69	0.42
NH_4_-N	0.64	0.85	0.66	0.58
${\text{NO}}_{3}^{-}$-N	–	0.30	0.56	–
Temperature	–	−0.25	–	−0.64
Moisture	–	–	–	–
**AOA**				
	7	14	29	57
pH	–	−0.42	−0.65	–
NH_3_-N	–	−0.28	–	−0.36
NH_4_-N	–	−0.28	–	−0.44
${\text{NO}}_{3}^{-}$-N	–	0.30	0.29	−0.45
Temperature	–	0.40	0.69	–
Moisture	–	−0.45	−0.25	–
**COM**				
	7	14	29	57
pH	–	−0.44	–	–
NH_3_-N	–	−0.62	−0.63	−0.68
NH_4_-N	–	−0.66	−0.66	−0.57
${\text{NO}}_{3}^{-}$-N	−0.33	−0.45	−0.73	−0.89
Temperature	−0.36	–	–	−0.40
Moisture	–	–	–	–

Dashes (–) indicate the correlation was non-significant (P > 0.05).

Within the 20HN700 treatment, AOB *amoA* gDNA growth began on day 1 and peaked on day 29 (Supplementary Figure 1). This coincided with the peak nitrification rate (day 1–29, 31.2 kg ${\text{NO}}_{3}^{-}$-N ha^−1^ day^−1^). Comparatively, AOB growth within the 8HN700 treatment began 14 days later and peaked at day 57. This also coincided with the peak nitrification rate of 19.2 kg ${\text{NO}}_{3}^{-}$-N ha^−1^ day^−1^ (from day 14–57). However, peak nitrification in the 8HN700 treatment was 38.6% less than the 20HN700 treatment (Supplementary Figure 2). This coincides with differences in AOB *amoA* gDNA accumulation rates over the same period (8HN700 44.8% less than the 20HN700 treatment). AOB *amoA* transcript abundance did not show this trend.

#### AOA

**Figure 2:**
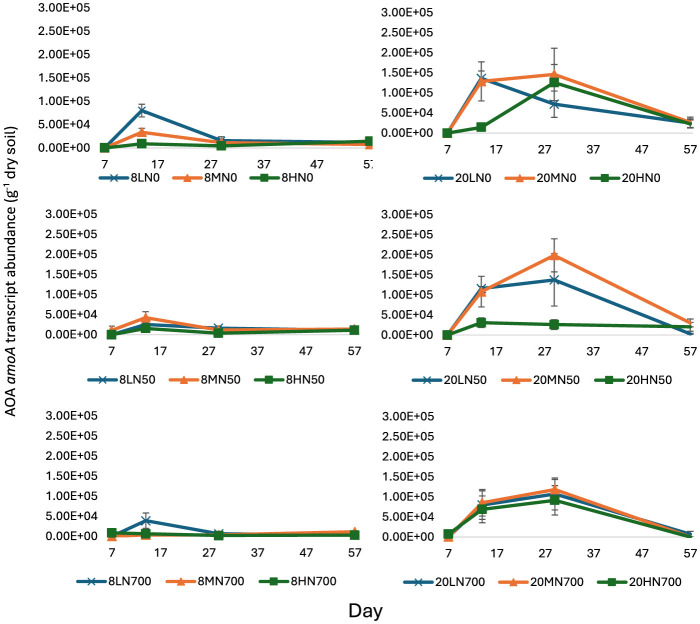
AOA *amoA* transcript abundance from day 7 to 57. Error bars represent the standard error of the mean (*n* = 4). 8, 8°C; 20, 20°C; L, low moisture (28.5% θ g); M, medium moisture (37.5% θg); H, high moisture (46.5% θ g); N0, no added nitrogen; N50, N applied at a rate of 50 kg N ha^−1^ as urea; N700, N applied at a rate of 700 kg N ha^−1^ as synthetic urine.

Throughout the trial, AOA *amoA* transcript abundance was strongly influenced by temperature (*P* < 0.001, v.r. = 71.81). A significant moisture effect (*P* = 0.003, v.r. = 6.52) was also observed, while the nitrogen effect was non-significant (*P* = 0.302; Figure 2).

At day 7, AOA *amoA* transcript was almost undetected. From day 14 to 29, AOA *amoA* transcript abundance was significantly higher in the 20°C treatments (Figure 2).

In the 20°C treatments, AOA *amoA* transcript abundance peaked at day 29 (except for 20LN0 and 20HN50), where the highest counts were 1.46 × 10^5^ g^−1^ dry soil (20MN0), 1.99 × 10^5^ g^−1^ dry soil (20MN50), and 1.18 × 10^5^ g^−1^ dry soil (20MN700) for 20N0, 20N50, and 20N700 treatments, respectively (Figure 2). In the 8°C treatments, AOA *amoA* transcript abundance peaked 15 days earlier, with 8.01 × 10^4^ g^−1^ dry soil (8LN0), 3.90 × 10^4^ g^−1^ dry soil (8MN50), and 4.24 × 10^4^ g^−1^ dry soil (8LN700) for 8N0, 8N50, and 8N700 treatments, respectively. Interestingly, the peak abundance in the 20N50 and 20N700 treatments was almost one order of magnitude higher than their 8°C counterparts (Figure 2).

At day 57, AOA *amoA* transcript abundance was significantly lower in the N700 treatments relative to the N50 and N0 treatments (*P* < 0.001). At peak abundance, high nitrogen-induced inhibition was higher in the 8°C treatments (51.3 vs. 21.3%, 90 vs. 19.1%, and 28.5 vs. 27.3% for the low, medium, and high moisture treatments, respectively).

AOA *amoA* transcript abundance shared a negative correlation with soil pH at days 14 and 29 (−0.42 and −0.65, respectively).

#### COM

**Figure 3:**
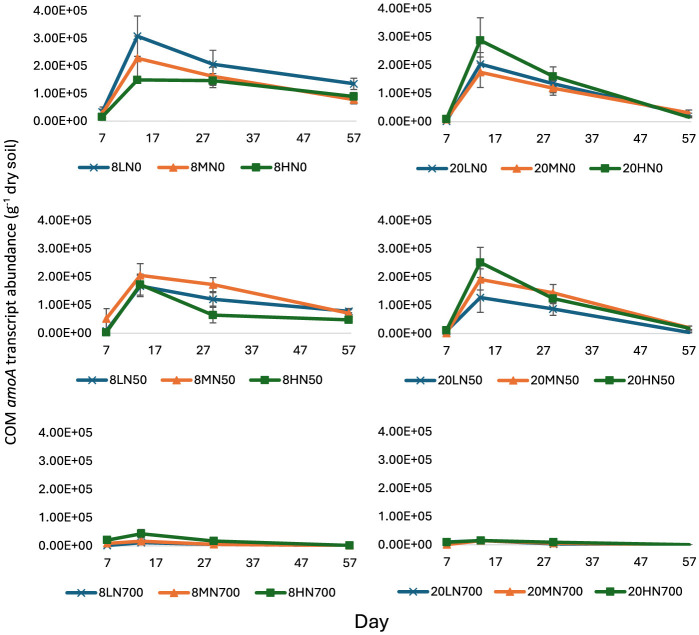
Comammox *Nitrospira amoA* transcript abundance from day 7 to 57. Error bars represent standard error of the mean (*n* = 4). 8, 8°C; 20, 20°C; L, low moisture (28.5% θ g); M, medium moisture (37.5% θ g); H, high moisture (46.5% θ g); N0, no added nitrogen; N50, N applied at a rate of 50 kg N ha^−1^ as urea; N700, N applied at a rate of 700 kg N ha^−1^ as synthetic urine.

Throughout the trial, nitrogen (*P* < 0.001, v.r. = 71.66) had the most influence on comammox *Nitrospira amoA* transcript abundance. Temperature had a minor effect (*P* = 0.03. v.r. = 5.01). While the moisture effect (*P* = 0.935) was calculated to be non-significant (Figure 3).

From day 14 onwards, comammox *Nitrospira amoA* transcript abundance was significantly lower in the N700 treatments (*P* < 0.001).

Except for 8HN0, comammox *Nitrospira amoA* transcript abundance peaked at day 14, where counts were highest in the 8LN0 (3.08 × 10^5^ g^−1^ dry soil), 20HN50 (2.52 × 10^5^ g^−1^ dry soil), and 8HN700 (4.27 × 10^4^ g^−1^ dry soil) for the respective N treatments (Figure 3). Peak comammox *Nitrospira amoA* transcript abundance in the N0 and N50 treatments was almost one order of magnitude higher than the N700 treatments.

Peak comammox *Nitrospira amoA* transcript abundance in the 8HN700 treatment was almost four times higher than the other 8N700 treatments (Figure 3).

Comammox *Nitrospira amoA* transcript abundance shared a strong negative correlation with soil ammonia and ammonium concentrations from day 14 to 57. At day 14, comammox *Nitrospira amoA* transcript abundance also shared a negative correlation with soil pH and soil nitrate concentrations (Table 1).

### Diversity analysis

#### AOB

A total of 2,770,824 high-quality AOB *amoA* transcript sequences were obtained from 72 samples. The sequencing analysis revealed that the AOB community was made up of 58 ASVs, grouped into five clades; *Nitrosospira* 3a.1 (81.5%), *Nitrosospira* 10/11 (8.42%), *Nitrosospira* 3b (1.69%), *Nitrosospira* 2/4 (0.826%), and *Nitrosomonas* (7.9%). ASV003 made up 80.7% of the AOB community (Supplementary Figure 3).

Temperature shared contrasting correlations with *Nitrosospira* 3a.1 and *Nitrosomonas*, with an *r*^2^ of −0.46 and 0.47, respectively (*P* < 0.0001; Supplementary Table 3).

*Nitrosomonas* also shared a significant positive correlation with soil nitrate and estimated ammonia concentration (*P* < 0.0001, *r*^2^ = 0.50, *P* = 0.0051, *r*^2^ = 0.33, respectively; Supplementary Table 3). Comparatively, the relative abundance of all *Nitrosospira* clusters were not positively correlated with soil ammonia or ammonium concentrations (Supplementary Table 3).

#### AOA

A total of 2,840,949 high-quality AOA *amoA* transcript sequences were obtained from 72 samples. The sequencing analysis revealed that the AOA community was made up of 22 ASVs, grouped into two clades: *Nitrososphaerales* (86.2%), and *Nitrosotaleales* (13.8%). The most abundant ASV, ASV011 made up 63.5% of the total community (Supplementary Figure 4). ASV002 was the only AOA ASV that was positively correlated with estimated ammonia availability (Supplementary Table 4). ASV002 showed 100% similarity to *Nitrosocomicus* sp.

**Figure 4:**
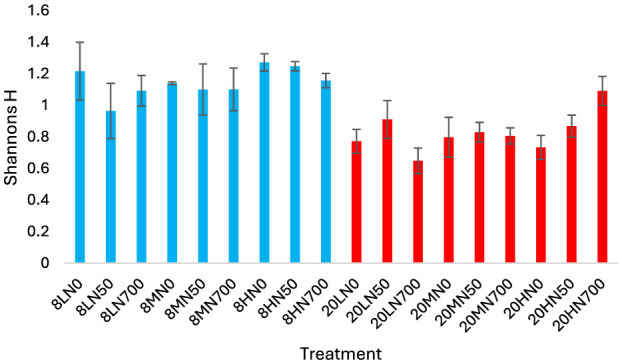
Shannons entropy of the AOA community in each treatment. Error bars represent standard error of the mean. 8, 8°C; 20, 20°C; L, low moisture (28.5% θ g); M, medium moisture (37.5% θ g); H, high moisture (46.5% θg); N0, no added nitrogen; N50, N applied at a rate of 50 kg N ha^−1^ as urea; N700, N applied at a rate of 700 kg N ha^−1^ as synthetic urine.

AOA community diversity was significantly higher in the 8°C treatments relative to the 20°C treatments (*P* < 0.001; Figure 4). No treatment effect was observed within each temperature block.

**Figure 5:**
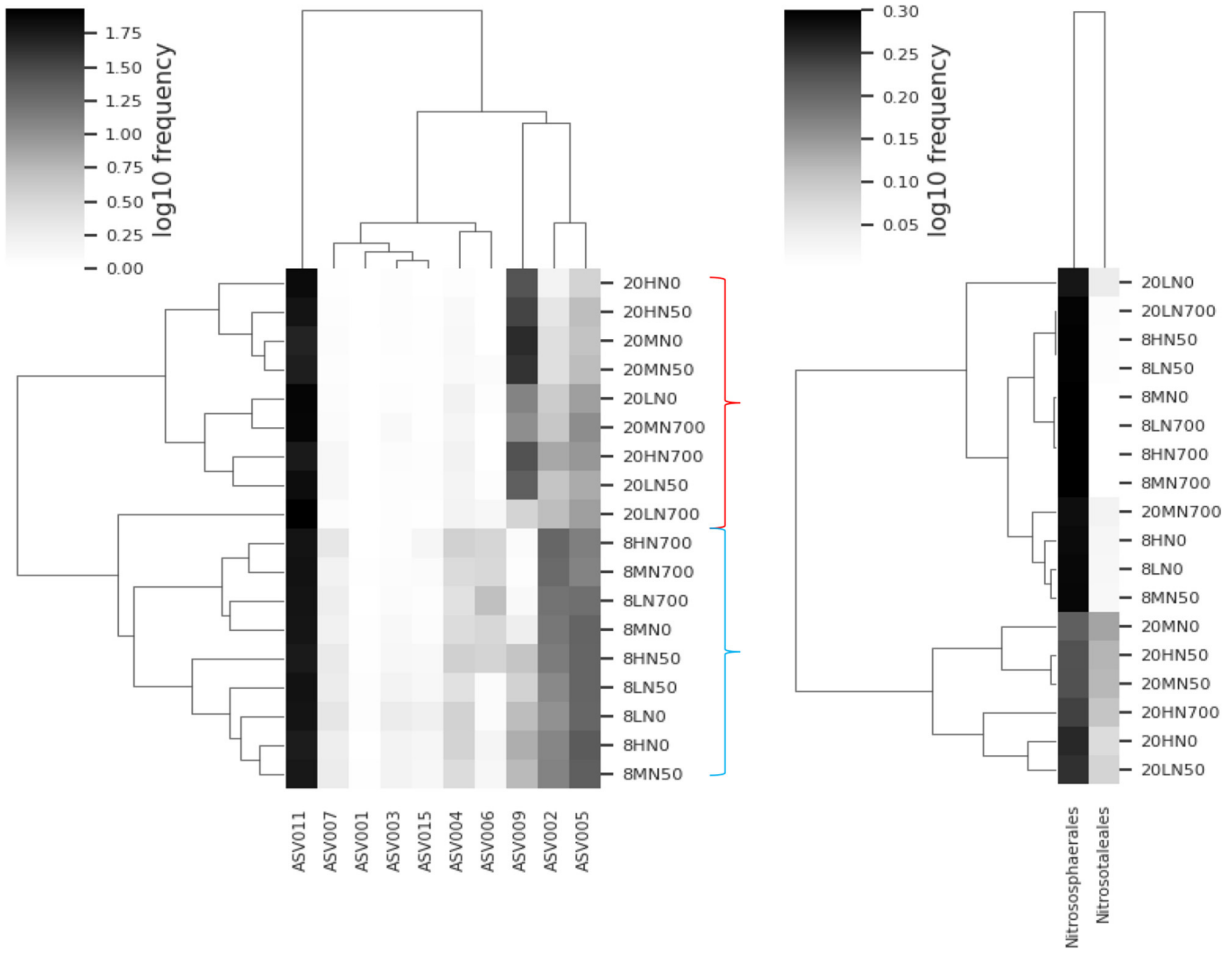
Heatmap of the common AOA ASVs **(left)** and the AOA clades within **(right)** each treatment. Relative abundance of clades is based on the associated ASVs shown in the phylogenetic tree (Supplementary Figure 4). Red bracket indicates the 20°C treatments. Blue bracket indicates the 8°C treatments. 8, 8°C; 20, 20°C; L, low moisture (28.5% θ g); M, medium moisture (37.5% θ g); H, high moisture (46.5% θg); N0, no added nitrogen; N50, N applied at a rate of 50 kg N ha^−1^ as urea; N700, N applied at a rate of 700 kg N ha^−1^ as synthetic urine.

Figure 5 shows that at 8°C, less abundant ASVs (members of *Nitrososphaerales*) made up a larger proportion of the AOA community. At 20°C, the relative abundance of the *Nitrosotaleales* clade significantly increased, while the proportion of ASVs associated with *Nitrosophaerales* decreased. Within the 20°C treatments, the relative abundance of *Nitrosotaleales* was reduced by synthetic urine. The relative abundance of ASV009 and ASV011 were positively correlated with temperature, while all other significant ASVs were negatively correlated (Supplementary Table 4). Similarly, ASV009 was the only ASV that was negatively correlated with pH and ammonium (Supplementary Table 4).

The Bray-Curtis analysis shows groupings of AOA beta diversity based on the two temperatures used (Supplementary Figure 13). Within the 8°C treatment, the application of synthetic urine seemed to also cause a community shift.

#### COM

The analysis of the 72 samples yielded 2,757,309 high-quality comammox *Nitrospira amoA* sequences. The comammox *Nitrospira* community was composed of 46 ASVs, divided into 3 clades. Clade B.2 was the most abundant, accounting for 78.6% of the population, followed by clade B.1 and clade A.2, which made up 21.3% and 0.021% of the population, respectively. Only 1 ASV, ASV005 was associated with comammox *Nitrospira* clade A.2 (Supplementary Figure 5).

Comammox *Nitrospira* alpha diversity was significantly affected by Temperature (*P* < 0.001), moisture (*P* = 0.002), and nitrogen (*P* < 0.001; Supplementary Figure 11). Moreover, comammox *Nitrospira* community diversity had a contrasting response to synthetic urine at 8 and 20°C. At 8°C, alpha diversity increased, while at 20°C, it decreased (Supplementary Figure 11).

The most abundant ASV, ASV002 (a member of clade B.2) was reduced under the 8N700 treatments. In comparison, the relative abundance of both ASV009 and ASV016 (members of clade B.1) increased under the 8N700 treatments (Supplementary Figure 12).

## Discussion

The contrasting response of AOA, AOB, and comammox *Nitrospira amoA* transcript abundance to temperature, moisture, and nitrogen investigated in this study gives novel insight into their respective roles in New Zealand's fertile dairy farm soils.

Similar to previous studies, AOB positively responded to the application of synthetic urine (Nicol et al., 2008; Di et al., 2009, 2014; Chisholm et al., 2024). Synthetic urine increased AOB *amoA* cDNA abundance to 1–3 orders of magnitude higher than AOA and comammox *Nitrospira amoA* transcript abundance. The relative response of AOB, plus the strong positive correlation between NH_3_-N, NH_4_-N, and AOB *amoA* transcript abundance, suggests that they are the main contributors to nitrification in a nitrogen-rich environment.

Peak AOB *amoA* gDNA abundance in the 8 and 20°C N700 treatments was similar (Supplementary Figure 1). However, at 20°C, the peak occurred 30 days earlier. Within the N700 treatments, AOB growth (AOB gDNA *amoA* abundance) followed a similar trend to ${\text{NO}}_{3}^{-}$ accumulation/nitrification rate (Supplementary Figures 1, 2). The AOB *amoA* transcript abundance did not follow the nitrate accumulation trend. This suggests that while the soil ammonia/ammonium concentration dictates the AOB *amoA* transcript response, ultimately, the AOB *amoA* gDNA community abundance is indicative of the soil nitrification rate.

The relative abundance of *Nitrosomonas* members was higher in the N700 treatments. This was more noticeable at 20°C (Supplementary Figure 9). All of the *Nitrosomonas* ASVs detected in this study were closely associated with *Nitrosomonas europaea*. This was expected as they: (i) possess a high ammonia saturation constant (Suzuki et al., 1974; Jiang and Bakken, 1999), (ii) are more tolerant to free nitrous acid than other AOB species (Liu et al., 2023), (iii) are more responsive to high ammonium availability after a period of starvation (Bollmann et al., 2005). However, it does raise the question: “why are *Nitrosospira* the dominant member of the AOB community?”. The answer is likely multifactorial. A possible partial explanation is that they are more tolerant to low pH, and are more suited to an environment with fluctuating ammonia availability (Groeneweg et al., 1994; Jiang and Bakken, 1999; Burton and Prosser, 2001; Terada et al., 2013). While *Nitrosomonas europaea* are more responsive to sudden and significant increases in ammonia availability, they are also less tolerant to low ammonia environments.

As stated previously, AOB *amoA* gDNA abundance was highest in the high moisture treatments. Di et al. (2014) also concluded that AOB *amoA* gDNA abundance responded positively to soil moisture. This was attributed to the presence of *nirK* in some AOB genomes. The *nirK* gene facilitates the production of nitrite reductase enzyme, which allows AOB to undertake a process known as nitrifier denitrification in oxygen limited environments (Wrage et al., 2001; Wrage-Mönnig et al., 2018). However, there was no significant variation in nitrate accumulation within the N700 treatments, despite the varying rate at which the concentration of ammonium (and estimated ammonia availability) decreased (Supplementary Figures 6, 7). This trend was particularly noticeable in the 20N700 treatments, most likely due to the relatively lower soil pH (Wrage-Mönnig et al., 2018). A possible explanation is reciprocal feeding within the AOB community, whereby ${\text{NO}}_{2}^{-}$ is reduced to NO and oxidized back to ${\text{NO}}_{2}^{-}$ with fluctuating O_2_ availability (Caranto and Lancaster, 2017).

Within the N0 and N50 treatments, the concentration of ${\text{NO}}_{3}^{-}$ was lower at high moisture contents, suggesting that traditional nitrifier denitrification rather than the proposed reciprocal feeding occurred.

As far as the authors are aware, this is the first study to investigate the effect of temperature on AOA activity and abundance in New Zealand soils.

Both AOA *amoA* transcript and gDNA abundance were significantly higher in the 20°C treatments (Figure 2, Supplementary Figure 8). Comparatively, temperature did not alter AOB peak transcript or gDNA *amoA* abundance. Taylor et al. (2017) showed that AOA and AOB were active across different temperature ranges. However, the two temperatures used in this study were within the range dominated by AOB activity/abundance (Taylor et al., 2017). Previous studies have suggested that for nitrification to be dominated by AOA, temperatures must exceed 30°C (Tourna et al., 2011; Lehtovirta-Morley et al., 2016b; Taylor et al., 2017). This exceeds all monthly average soil temperatures in New Zealand. It is also worth noting that the nitrification potential outlined in Taylor et al. (2017) was without nitrogen fertilizer application. Many AOA possess a high substrate affinity for ammonia (Offre et al., 2014), which allows them to thrive in an oligotrophic/nutrient deficient environment (Lehtovirta-Morley et al., 2016b; Herbold et al., 2017; Ouyang et al., 2017). Because of this, their relative growth potential is limited under nitrogen-amended soils. Furthermore, AOA *amoA* transcript and gDNA growth was inhibited in the N700 treatments.

AOA may have significantly contributed to ammonia oxidation in the 50 kg ha^−1^ urea amended soils at 20°C. However, AOA *amoA* transcriptional response occurred 14 days after peak AOB *amoA* transcript abundance. This was also after much of the soil ammonium was consumed (Supplementary Figure 6). This indicates that AOA only responded after the soil ammonium concentration became non-limiting.

However, the relative abundance of AOA ASV002 increased in the N700 treatments. ASV002 shared 100% identity with *Candidatus Nitrosocosmicus* franklandus. *Ca. N*. franklandus has been characterized as having a comparable ammonia affinity to soil borne AOB and responsive to high urea concentration (Lehtovirta-Morley et al., 2016a). Lehtovirta-Morley et al. (2016a) showed that *Ca. N*. franklandus had an optimum temperature of 40°C, and did not grow at 25°C. ASV002 shared a negative correlation with soil temperature, which suggests that in soil borne *Nitrosocosmicus* may be tolerant to a wider range of temperatures. The presence and relative increase of *Nitrosocosmicus* AOA in a high nitrogen environment poses an interesting contrast from conclusions derived from Di et al. (2009). However, as the AOA *amoA* transcript and gDNA abundance was significantly inhibited in the N700 treatments, it is unlikely that the overall AOA community significantly contribute to nitrification in New Zealand dairy farm soils.

Temperature was the sole treatment to affect soil pH (Supplementary Table 5). At a higher temperature, soil pH was significantly lower. Because of this, the observed temperature effect may be confounded by a soil pH effect (Lu et al., 2018). Both of these influence AOA activity and abundance (Herbold et al., 2017; Gubry-Rangin et al., 2017; Taylor et al., 2017).

Gubry-Rangin et al. (2017) observed that the AOA temperature response was pH dependant. In a low soil pH environment, optimum temperature was at 20°C, while in neutral soils, it was 30°C. It was concluded that two abundant acidic clusters (*Nitrososphaera* and *Nitrosotalea*) may prefer lower temperatures, relative to neutrophilic thaumarchaeotal clusters.

The relative abundance of AOA *amoA* ASV009 increased in all 20°C treatments, while the relative abundance of all other ASVs decreased (ASV009 is a member of *Nitrosotaleales*).

Previous studies have reported that *Nitrosotaleales* can thrive at a low soil pH. Their optimal growth temperature is one of the lowest recorded at 25°C (Lehtovirta-Morley et al., 2011). Comparatively, the optimum temperature for *Nitrososphaeales* (*Nitrososphaeales viennensis*) was 35°C (Tourna et al., 2011). This provides a possible explanation for the relative increase in *Nitrosotaleales*, while the relative abundance of other AOA community members decreased or remained unchanged. It is likely that the positive relationship between AOA activity and temperature is due to the relative increase of the *Nitrosotaleales* clade, while the *Nitrososphaeales* clade had a smaller effect.

The relative abundance of *Nitrosotaleales* decreased under the synthetic urine treatments at 20°C. A possible explanation is inhibition by free nitrous acid. Lehtovirta-Morley et al. (2011) demonstrated that *Nitrosotalea devanterra* was inhibited by 2.53 μM free nitrous acid (40 μM nitrite at pH 4.5).

Comammox *Nitrospira amoA* transcript and gDNA abundance did not respond to soil moisture. This suggests that the moisture-comammox *Nitrospira* association found in Chisholm et al. (2023) is more complex than previously thought. It is possible that the high moisture found in Chisholm et al. (2023) facilitated an interaction between comammox *Nitrospira* and another microorganism, such as anammox (Vilardi et al., 2023; Guo et al., 2024). Or the high moisture facilitated a change in environment that was undetected. Chisholm et al. (2023) sampled a diverse range of terrestrial ecosystems. These sites contained a wide variety of plant species and soil types which could be indirectly linked to soil moisture. Wang et al. (2021) indicated that comammox *Nitrospira* community composition was significantly influenced by plant type. Comammox *Nitrospira* clade B have the ability to oxidize formate and other metabolites suggestive of a mixotrophic nature (Palomo et al., 2018; Palatinszky et al., 2024). Formate is produced from the decomposition of organic matter, plant root exudation or excreted as a microbial metabolic byproduct (Adeleke et al., 2017). Therefore, future studies should carefully consider biotic interactions to explain the relative distribution of comammox *Nitrospira* clade B.

Despite the presence of genes suggestive of a microaerophilic preference in the comammox *Nitrospira* genome (Palomo et al., 2019), the results presented in Masta et al. (2022) indicate that comammox *Nitrospira amoA* abundance remain unchanged with changing oxygen availability, further indicating that this relationship is more complex than previously thought. Clearly, additional research is required to understand this phenomenon.

Similarly to AOB, comammox *Nitrospira* did not respond to temperature. This may be because comammox *Nitrospira* obtained the ability to oxidize ammonia through a horizontal gene transfer event with β-AOB, indicating that their ammonia monooxygenase enzymes are physiologically similar (Palomo et al., 2018). However, Feng et al. (2022) concluded that the comammox *Nitrospira* abundance shared a negative correlation with soil temperature. This was attributed to either a low optimum temperature for clade A.2 or the decrease in soil pH caused by a temperature-induced higher nitrification rate. Based on enriched and isolated cultures, the optimum temperature for comammox *Nitrospira* is 37 and 23°C for *N. Inopinata* and *N. Nitrosa*, respectively (Van Kessel et al., 2015; Kits et al., 2017). However, all current isolated and enriched cultures were obtained from an aquatic environment and are members of clade A.1. Whereas the community in this study was dominated by clade B and derived from soil.

It is well established that the relative abundance of comammox *Nitrospira* clades is determined by key environmental parameters (Wang et al., 2021; Gao et al., 2023). Physiochemical properties like soil pH are considered niche separating (Wang et al., 2021).

It is also possible that comammox *Nitrospira* was limited by other environmental conditions within this study. Therefore, their response to desirable conditions was limited. A possible example of this could be soil ammonia availability.

Comammox *Nitrospira* transcript *amoA* abundance was negatively correlated with estimated ammonia, ammonium, and nitrate concentrations (Table 1). Isolated cultures suggest that comammox *Nitrospira* prefer an oligotrophic environment (Kits et al., 2017) as genomic analysis revealed that they possess MEP-type ammonia transporters (Palomo et al., 2018). These ammonia transporters have a high affinity (micromolar range) and low uptake capacity (Walter et al., 2008). The genomic analysis also revealed an extra non-operon *amoC* gene, which is responsible for adaption to low nitrosative stress and ammonia starvation conditions (Palomo et al., 2018; Koch et al., 2019; Xu et al., 2020). The lack of response to changes in soil moisture suggest that there is an additional abiotic or biotic parameter that has not been considered.

The disparity between comammox *Nitrospira amoA* transcript and *amoA* gDNA abundance trend indicate that comammox may have gained energy from a source other than ammonia oxidation. Kits et al. (2017) showed that comammox *Nitrospira inopinata* have a lower nitrite affinity than canonical *Nitrospira* (canonical *NitrospiraK*_m_: 6–27 μM ${\text{NO}}_{2}^{-}$ vs. Comammox *K*_m(app)_: 449.2 μM ${\text{NO}}_{2}^{-}$). Therefore, it is possible that comammox *Nitrospira* preferentially undertake the second step of nitrification in a high nitrogen environment, while acting similar to canonical ammonia oxidisers in a low nitrogen environment.

As far as the authors are aware, this is the first paper to find comammox *Nitrospira* clade A.2 in New Zealand soils. Previous papers found that the community solely consisted of clade B (Hsu et al., 2022; Chisholm et al., 2023, 2024; Shah et al., 2024). The discovery of clade A.2 rules out geographical isolation as a potential explanation for their absence in previous studies. Therefore, a different explanation must be explored. Previous papers suggests that soil pH is a possible driver of clade A.2 abundance and comammox *Nitrospira* niche specificity (Wang et al., 2021). However, no other studies have found the almost complete dominance of clade B. These results indicate that there is another, previously unidentified environmental parameter (either biotic or abiotic) that heavily influences the abundance of comammox *Nitrospira* clade B in terrestrial ecosystems. However, based on our current understanding, it is fair to assume that comammox *Nitrospira* clade B do not significantly contribute to nitrification in New Zealand soils.

Overall, the results presented in this paper support the current paradigm that AOB are the major contributor to nitrification in a nitrogen-rich soil. While both the AOA and comammox *Nitrospira* communities are inhibited by high ammonia availability. It is also possible that, while capable of ammonia oxidation, both AOA and comammox *Nitrospira* are capable of a wide range of metabolic pathways that allow them to thrive in a low nitrogen environment. However, the relative abundance of *Nitrosocosmicus* AOA did increase in response to the application of synthetic urine. However, they were not a major member of the AOA community. Their presence and relative increase pose an interesting question: “are there nutrient rich environments in which they are the dominant ammonia oxidiser?”.

## Conclusion

Ammonia oxidizing bacteria dominated soil treated with synthetic urine at low, medium, and high soil moisture, and at high and low temperature. AOB *amoA* gDNA abundance was highest in the high moisture treatments. This was attributed to the presence of *nirK* in some AOB genomes. The *nirK* gene facilitates the production of nitrite reductase enzyme, which allows AOB to undertake a process known as nitrifier denitrification. However, soil ammonium and nitrate concentrations suggest that the AOB present in the synthetic urine treatments participated in reciprocal feeding, whereby ${\text{NO}}_{2}^{-}$ is reduced to NO and oxidized back to ${\text{NO}}_{2}^{-}$ with fluctuating O_2_ availability.

Ammonia oxidizing archaea *amoA* abundance and community composition was strongly influenced by soil temperature. At 20°C, AOA *amoA* peak transcript abundance averaged over 1 order of magnitude higher. ASV002 (a member of the *Nitrosocosmicus* clade) was positively correlated to ammonium and estimated soil ammonia concentrations. The presence and relative increase of *Nitrosocosmicus* AOA in a high nitrogen environment poses an interesting contrast from current scientific opinion.

Contrasting to a previous study, the abundance of comammox *Nitrospira* was not positively correlated with soil moisture. This suggests that the association is more complex than previously thought. Further research is required to determine the drivers of comammox *Nitrospira* abundance in a high moisture environment. The majority of the comammox *Nitrospira* community was associated with clade B, with a small clade A.2 component. As far as the authors are aware, this is the first New Zealand paper to identify sequences associated with clade A.2. Overall, these results indicate that AOB are the main drivers of nitrification in New Zealand dairy farm soils.

## Data Availability

The datasets presented in this study can be found in online repositories. The names of the repository/repositories and accession number(s) can be found in the article/Supplementary material.

## References

[B1] Adeleke R. Nwangburuka C. Oboirien B. (2017). Origins, roles and fate of organic acids in soils: a review. S. Afr. J. Bot. 108, 393–406. 10.1016/j.sajb.2016.09.002

[B2] Blakemore L. C. (1987). Methods for chemicalanalysis of soils. NZ Soil Bureau Sci. Rep. 80, 72–76.

[B3] Bokulich N. A. Subramanian S. Faith J. J. Gevers D. Gordon J. I. Knight R.. (2013). Quality-filtering vastly improves diversity estimates from Illumina amplicon sequencing. Nat. Methods 10, 57–59. 10.1038/nmeth.2276 23202435 PMC3531572

[B4] Bollmann A. Schmidt I. Saunders A. M. Nicolaisen M. H. (2005). Influence of starvation on potential ammonia-oxidizing activity and amoA mRNA levels of *Nitrosospira briensis*. Appl. Environ. Microbiol. 71, 1276–1282. 10.1128/AEM.71.3.1276-1282.2005 15746329 PMC1065156

[B5] Bolyen E. Rideout J. R. Dillon M. R. Bokulich N. A. Abnet C. C. Al-Ghalith G. A.. (2019). Reproducible, interactive, scalable and extensible microbiome data science using QIIME 2. Nat. Biotechnol. 37, 852–857. 10.1038/s41587-019-0209-9 31341288 PMC7015180

[B6] Brown I. C. (1943). A rapid method of determining exchangeable hydrogen and total exchangeable bases of soils. Soil Sci. 56, 353–358. 10.1097/00010694-194311000-00004

[B7] Burton S. A. Prosser J. I. (2001). Autotrophic ammonia oxidation at low pH through urea hydrolysis. Appl. Environ. Microbiol. 67, 2952–2957. 10.1128/AEM.67.7.2952-2957.2001 11425707 PMC92966

[B8] Callahan B. J. McMurdie P. J. Rosen M. J. Han A. W. Johnson A. J. A. Holmes S. P.. (2016). DADA2: High-resolution sample inference from Illumina amplicon data. Nat. Methods 13, 581–583. 10.1038/nmeth.3869 27214047 PMC4927377

[B9] Cameron K. C. Di H. J. Moir J. L. (2013). Nitrogen losses from the soil/plant system: a review. Ann. Appl. Biol. 162, 145–173. 10.1111/aab.12014

[B10] Caranto J. D. Lancaster K. M. (2017). Nitric oxide is an obligate bacterial nitrification intermediate produced by hydroxylamine oxidoreductase. Proc. Nat. Acad. Sci. 114, 8217–8222. 10.1073/pnas.1704504114 28716929 PMC5547625

[B11] Chisholm C. Di H. Cameron K. Podolyan A. Shen J. Zhang L.. (2024). Contrasting response of comammox *Nitrospira*, ammonia oxidising bacteria, and archaea to soil pH and nitrogen inputs. Sci. Total Environ. 924:171627. 10.1016/j.scitotenv.2024.171627 38471592

[B12] Chisholm C. Di H. J. Cameron K. Podolyan A. Shah A. Hsu L.. (2023). Soil moisture is a primary driver of comammox *Nitrospira* abundance in New Zealand soils. Sci. Total Environ. 858:159961. 10.1016/j.scitotenv.2022.159961 36343813

[B13] Clough T. J. Ledgard S. F. Sprosen M. S. Kear M. J. (1998). Fate of 15 N labelled urine on four soil types. Plant Soil 199, 195–203. 10.1023/A:1004361009708

[B14] Daims H. Lebedeva E. V. Pjevac P. Han P. Herbold C. Albertsen M.. (2015). Complete nitrification by *Nitrospira* bacteria. Nature 528, 504–509. 10.1038/nature16461 26610024 PMC5152751

[B15] Di H. J. Cameron K. C. Podolyan A. Robinson A. (2014). Effect of soil moisture status and a nitrification inhibitor, dicyandiamide, on ammonia oxidizer and denitrifier growth and nitrous oxide emissions in a grassland soil. Soil Biol. Biochem. 73, 59–68. 10.1016/j.soilbio.2014.02.011

[B16] Di H. J. Cameron K. C. Shen J.-P. Winefield C. S. O'Callaghan M. Bowatte S.. (2010). Ammonia-oxidizing bacteria and archaea grow under contrasting soil nitrogen conditions. FEMS Microbiol. Ecol. 72, 386–394. 10.1111/j.1574-6941.2010.00861.x 20370827

[B17] Di H. J. Cameron K. C. Shen J. P. Winefield C. S. O'Callaghan M. Bowatte S.. (2009). Nitrification driven by bacteria and not archaea in nitrogen-rich grassland soils. Nat. Geosci. 2, 621–624. 10.1038/ngeo613

[B18] Dixon P. (2003). VEGAN, a package of R functions for community ecology. J. Veg. Sci. 14, 927–930. 10.1111/j.1654-1103.2003.tb02228.x

[B19] Feng M. He Z.-Y. Fan J. Ge A.-H. Jin S. Lin Y.. (2022). Temperature has a strong impact on the abundance and community structure of comammox *Nitrospira* in an Ultisol. J. Soils Sediments 22, 2593–2603. 10.1007/s11368-022-03261-5

[B20] Francis C. A. Beman J. M. Kuypers M. M. M. (2007). New processes and players in the nitrogen cycle: the microbial ecology of anaerobic and archaeal ammonia oxidation. ISME J. 1, 19–27. 10.1038/ismej.2007.8 18043610

[B21] Francis C. A. Roberts K. J. Beman J. M. Santoro A. E. Oakley B. B. (2005). Ubiquity and diversity of ammonia-oxidizing archaea in water columns and sediments of the ocean. Proc. Nat. Acad. Sci. 102, 14683–14688. 10.1073/pnas.0506625102 16186488 PMC1253578

[B22] Gao F. Li Y. Fan H. Xue J. Yao H. (2023). Main environmental drivers of abundance, diversity and community structure of comammox *Nitrospira* in paddy soils. Pedosphere 33, 808–818. 10.1016/j.pedsph.2022.06.061

[B23] Groeneweg J. Sellner B. Tappe W. (1994). Ammonia oxidation in nitrosomonas at NH_3_ concentrations near km: effects of pH and temperature. Water Res. 28, 2561–2566. 10.1016/0043-1354(94)90074-4

[B24] Gubry-Rangin C. Novotnik B. Mandič-Mulec I. Nicol G. W. Prosser J. I. (2017). Temperature responses of soil ammonia-oxidising archaea depend on pH. Soil Biol. Biochem. 106, 61–68. 10.1016/j.soilbio.2016.12.007

[B25] Guo Z. Ma X. S. Ni S.-Q. (2024). Journey of the swift nitrogen transformation: unveiling comammox from discovery to deep understanding. Chemosphere 358:142093. 10.1016/j.chemosphere.2024.142093 38679176

[B26] Gustafsson J. P. (2011). Visual MINTEQ 3.0 User Guide. Stockholm: KTH, Department of Land and Water Recources, 550.

[B27] Harrell Jr F. E. Harrell Jr M. F. E. (2019). Package ‘hmisc.' CRAN2018 2019, 235–236.

[B28] Herbold C. W. Lehtovirta-Morley L. E. Jung M.-Y. Jehmlich N. Hausmann B. Han P.. (2017). Ammonia-oxidising archaea living at low pH: insights from comparative genomics. Environ. Microbiol. 19, 4939–4952. 10.1111/1462-2920.13971 29098760 PMC5767755

[B29] Hewitt A. E. (2010). New Zealand soil classification. Landcare Res. Sci. Ser.

[B30] Hsu P.-C. Di H. Cameron K. Podolyan A. Chau H. Luo J.. (2022). Comammox Nitrospira Clade B is the most abundant complete ammonia oxidizer in a dairy pasture soil and inhibited by dicyandiamide and high ammonium concentrations. Front. Microbiol. 13:1048735. 10.3389/fmicb.2022.1048735 36578577 PMC9791190

[B31] Huelsenbeck J. P. Ronquist F. (2001). MRBAYES: Bayesian inference of phylogenetic trees. Bioinformatics 17, 754–755. 10.1093/bioinformatics/17.8.754 11524383

[B32] Hunter J. D. (2007). Matplotlib: a 2D graphics environment. Comput. Sci. Eng. 9, 90–95. 10.1109/MCSE.2007.55

[B33] Jiang Q. Q. Bakken L. R. (1999). Comparison of *Nitrosospira* strains isolated from terrestrial environments. FEMS Microbiol. Ecol. 30, 171–186. 10.1111/j.1574-6941.1999.tb00646.x 10508942

[B34] Kits K. D. Sedlacek C. J. Lebedeva E. V. Han P. Bulaev A. Pjevac P.. (2017). Kinetic analysis of a complete nitrifier reveals an oligotrophic lifestyle. Nature 549, 269–272. 10.1038/nature23679 28847001 PMC5600814

[B35] Koch H. van Kessel M. A. Lücker S. (2019). Complete nitrification: insights into the ecophysiology of comammox *Nitrospira*. Appl. Microbiol. Biotechnol. 103, 177–189. 10.1007/s00253-018-9486-3 30415428 PMC6311188

[B36] Könneke M. Bernhard A. E. José R. Walker C. B. Waterbury J. B. Stahl D. A.. (2005). Isolation of an autotrophic ammonia-oxidizing marine archaeon. Nature 437, 543–546. 10.1038/nature03911 16177789

[B37] Lehtovirta-Morley L. E. Ross J. Hink L. Weber E. B. Gubry-Rangin C. Thion C.. (2016a). Isolation of ‘Candidatus *Nitrosocosmicus* franklandus', a novel ureolytic soil archaeal ammonia oxidiser with tolerance to high ammonia concentration. FEMS Microbiol. Ecol. 92:fiw057. 10.1093/femsec/fiw057 26976843 PMC4830249

[B38] Lehtovirta-Morley L. E. Sayavedra-Soto L. A. Gallois N. Schouten S. Stein L. Y. Prosser J. I.. (2016b). Identifying potential mechanisms enabling acidophily in the ammonia-oxidizing archaeon “Candidatus *Nitrosotalea devanaterra*.” *Appl. Environ*. Microbiol. 82, 2608–2619. 10.1128/AEM.04031-15 26896134 PMC4836417

[B39] Lehtovirta-Morley L. E. Stoecker K. Vilcinskas A. Prosser J. I. Nicol G. W. (2011). Cultivation of an obligate acidophilic ammonia oxidizer from a nitrifying acid soil. Proc. Nat. Acad. Sci. 108, 15892–15897. 10.1073/pnas.1107196108 21896746 PMC3179093

[B40] Li C. He Z.-Y. Hu H.-W. He J.-Z. (2022). Niche specialization of comammox *Nitrospira* in terrestrial ecosystems: Oligotrophic or copiotrophic? Crit. Rev. Environ. Sci. Technol. 53, 161–176. 10.1080/10643389.2022.2049578

[B41] Li C. Hu H.-W. Chen Q.-L. Chen D. He J.-Z. (2019). Comammox *Nitrospira* play an active role in nitrification of agricultural soils amended with nitrogen fertilizers. Soil Biol. Biochem. 138:107609. 10.1016/j.soilbio.2019.10760936578577

[B42] Liu Y. Zhu Y. Wu D. Wang Z. Wang Y. Wang G.. (2023). Effect of free nitrous acid on nitritation process: microbial community, inhibitory kinetics, and functional biomarker. Bioresour. Technol. 371:128595. 10.1016/j.biortech.2023.128595 36634879

[B43] Lu X. Nicol G. W. Neufeld J. D. (2018). Differential responses of soil ammonia-oxidizing archaea and bacteria to temperature and depth under two different land uses. Soil Biol. Biochem. 120, 272–282. 10.1016/j.soilbio.2018.02.017

[B44] Masta M. Espenberg M. Gadegaonkar S. S. Pärn J. Sepp H. Kirsimäe K.. (2022). Integrated isotope and microbiome analysis indicates dominance of denitrification in N2O production after rewetting of drained fen peat. Biogeochemistry 161, 119–136. 10.1007/s10533-022-00971-3

[B45] Nicol G. W. Leininger S. Schleper C. Prosser J. I. (2008). The influence of soil pH on the diversity, abundance and transcriptional activity of ammonia oxidizing archaea and bacteria. Environ. Microbiol. 10, 2966–2978. 10.1111/j.1462-2920.2008.01701.x 18707610

[B46] Offre P. Kerou M. Spang A. Schleper C. (2014). Variability of the transporter gene complement in ammonia-oxidizing archaea. Trends Microbiol. 22, 665–675. 10.1016/j.tim.2014.07.007 25169021

[B47] Olsen S. R. (1954). Estimation of Available Phosphorus in Soils by Extraction with Sodium Bicarbonate. Washington, DC: US Department of Agriculture.

[B48] Orellana L. H. Chee-Sanford J. C. Sanford R. A. Löffler F. E. Konstantinidis K. T. (2018). Year-round shotgun metagenomes reveal stable microbial communities in agricultural soils and novel ammonia oxidizers responding to fertilization. Appl. Environ. Microbiol. 84:e01646-17. 10.1128/AEM.01646-17 29101194 PMC5752871

[B49] Ouyang Y. Norton J. M. Stark J. M. (2017). Ammonium availability and temperature control contributions of ammonia oxidizing bacteria and archaea to nitrification in an agricultural soil. Soil Biol. Biochem. 113, 161–172. 10.1016/j.soilbio.2017.06.010

[B50] Palatinszky M. Herbold C. W. Sedlacek C. J. Pühringer D. Kitzinger K. Giguere A. T.. (2024). Growth of complete ammonia oxidizers on guanidine. Nature 633, 646–653. 10.1038/s41586-024-07832-z 39143220 PMC11410670

[B51] Palomo A. Dechesne A. Smets B. F. (2019). Genomic profiling of *Nitrospira* species reveals ecological success of comammox *Nitrospira. bioRxiv* 612226. 10.1101/612226 PMC9714041 36451244

[B52] Palomo A. Pedersen A. G. Fowler S. J. Dechesne A. Sicheritz-Pontén T. Smets B. F.. (2018). Comparative genomics sheds light on niche differentiation and the evolutionary history of comammox *Nitrospira*. ISME J. 12, 1779–1793. 10.1038/s41396-018-0083-3 29515170 PMC6018701

[B53] Prosser J. I. Nicol G. W. (2008). Relative contributions of archaea and bacteria to aerobic ammonia oxidation in the environment. Environ. Microbiol. 10, 2931–2941. 10.1111/j.1462-2920.2008.01775.x 18973620

[B54] Prosser J. I. Nicol G. W. (2012). Archaeal and bacterial ammonia-oxidisers in soil: the quest for niche specialisation and differentiation. Trends Microbiol. 20, 523–531. 10.1016/j.tim.2012.08.001 22959489

[B55] Rayment G. Higginson F. R. (1992). Australian Laboratory Handbook of Soil and Water Chemical Methods. Sydney, NSW: Inkata Press.

[B56] Rotthauwe J.-H. Witzel K.-P. Liesack W. (1997). The ammonia monooxygenase structural gene amoA as a functional marker: molecular fine-scale analysis of natural ammonia-oxidizing populations. Appl. Environ. Microbiol. 63, 4704–4712. 10.1128/aem.63.12.4704-4712.1997 9406389 PMC168793

[B57] Rütting T. Schleusner P. Hink L. Prosser J. I. (2021). The contribution of ammonia-oxidizing archaea and bacteria to gross nitrification under different substrate availability. Soil Biol. Biochem. 160:108353. 10.1016/j.soilbio.2021.10835334661841

[B58] Shah A. S. Hsu P.-C. L. Chisholm C. Podolyan A. Cameron K. Luo J.. (2024). Nitrification inhibitor chlorate and nitrogen substrates differentially affect comammox *Nitrospira* in a grassland soil. Front. Microbiol. 15:1392090. 10.3389/fmicb.2024.1392090 38808273 PMC11130707

[B59] Suzuki I. Dular U. Kwok S. (1974). Ammonia or ammonium ion as substrate for oxidation by *Nitrosomonas europaea* cells and extracts. J. Bacteriol. 120, 556–558. 10.1128/jb.120.1.556-558.1974 4422399 PMC245802

[B60] Takahashi Y. Fujitani H. Hirono Y. Tago K. Wang Y. Hayatsu M.. (2020). Enrichment of comammox and nitrite-oxidizing *Nitrospira* from acidic soils. Front. Microbiol. 11:1737. 10.3389/fmicb.2020.01737 32849373 PMC7396549

[B61] Taylor A. E. Giguere A. T. Zoebelein C. M. Myrold D. D. Bottomley P. J. (2017). Modeling of soil nitrification responses to temperature reveals thermodynamic differences between ammonia-oxidizing activity of archaea and bacteria. ISME J. 11, 896–908. 10.1038/ismej.2016.179 27996979 PMC5364361

[B62] Terada A. Sugawara S. Yamamoto T. Zhou S. Koba K. Hosomi M.. (2013). Physiological characteristics of predominant ammonia-oxidizing bacteria enriched from bioreactors with different influent supply regimes. Biochem. Eng. J. 79, 153–161. 10.1016/j.bej.2013.07.012

[B63] Tourna M. Stieglmeier M. Spang A. Könneke M. Schintlmeister A. Urich T.. (2011). Nitrososphaera viennensis, an ammonia oxidizing archaeon from soil. Proc. Nat. Acad. Sci. 108, 8420–8425. 10.1073/pnas.1013488108 21525411 PMC3100973

[B64] Van Kessel M. A. Speth D. R. Albertsen M. Nielsen P. H. Op den Camp H. J. Kartal B.. (2015). Complete nitrification by a single microorganism. Nature 528, 555–559. 10.1038/nature16459 26610025 PMC4878690

[B65] Vilardi K. Cotto I. Bachmann M. Parsons M. Klaus S. Wilson C.. (2023). Co-occurrence and cooperation between comammox and anammox bacteria in a full-scale attached growth municipal wastewater treatment process. Environ. Sci. Technol. 57, 5013–5023. 10.1021/acs.est.2c09223 36913533 PMC10061930

[B66] VSN International (2022). Genstat for Windows 22nd Edition [Internet]. Genstat.co.uk. Available at: https://vsni.co.uk/software/genstat/

[B67] Walter B. Küspert M. Ansorge D. Krämer R. Burkovski A. (2008). Dissection of ammonium uptake systems in *Corynebacterium glutamicum*: mechanism of action and energetics of AmtA and AmtB. J. Bacteriol. 190, 2611–2614. 10.1128/JB.01896-07 18245289 PMC2293182

[B68] Wang D.-Q. Zhou C.-H. Nie M. Gu J.-D. Quan Z.-X. (2021). Abundance and niche specificity of different types of complete ammonia oxidizers (comammox) in salt marshes covered by different plants. Sci. Total Environ. 768:144993. 10.1016/j.scitotenv.2021.144993 33736320

[B69] Wrage N. Velthof G. L. van Beusichem M. L. Oenema O. (2001). Role of nitrifier denitrification in the production of nitrous oxide. Soil Biol. Biochem. 33, 1723–1732. 10.1016/S0038-0717(01)00096-7

[B70] Wrage-Mönnig N. Horn M. A. Well R. Müller C. Velthof G. Oenema O.. (2018). The role of nitrifier denitrification in the production of nitrous oxide revisited. Soil Biol. Biochem. 123, A3–A16. 10.1016/j.soilbio.2018.03.020

[B71] Xu S. Wang B. Li Y. Jiang D. Zhou Y. Ding A.. (2020). Ubiquity, diversity, and activity of comammox *Nitrospira* in agricultural soils. Sci. Total Environ. 706:135684. 10.1016/j.scitotenv.2019.135684 31862588

[B72] Zhao Z. Huang G. He S. Zhou N. Wang M. Dang C.. (2019). Abundance and community composition of comammox bacteria in different ecosystems by a universal primer set. Sci. Total Environ. 691, 146–155. 10.1016/j.scitotenv.2019.07.131 31319252

