# Supplementary material for: Transcriptional activity of ammonia oxidisers in response to soil temperature, moisture and nitrogen amendment
Source: Front Microbiol. 2025 Jan 15;15:1466991. doi: 10.3389/fmicb.2024.1466991 (PMC11776869; doi:10.3389/fmicb.2024.1466991)
Supplement: Supplementary file 1 [file Presentation_1.pdf]

Supplementary Table 1. Soil chemical properties of the soil prior to treatment application.

	pH	Organic C (g kg^{-1})	Total N (g kg^{-1})	Olsen P (mg kg^{-1})	CEC (cmol$_c$ kg^{-1})	Exch Mg (cmol$_c$ kg^{-1})	Exch K (cmol$_c$ kg^{-1})	Exch Ca (cmol$_c$ kg^{-1})	Exch Na (cmol$_c$ kg^{-1})	Base Saturation (%)
Soil	5.8	4.8	0.28	42	16	1.21	0.67	6.3	0.23	51.1

Supplementary Table 2. Amplification conditions used for comammox, AOA and AOB *amoA* qPCR.

Target gene	Primer name	Primer sequence (5'-3')	Amplification conditions	Reference
Comammox *amoA*	comamoA F<br>comamoA R	AGGNGAYTGGGAYTTCTGG<br>CGGACAWABRTGAABCCCAT	95°C 2 min, 40 cycles 95°C 15 sec, 55°C 60 sec, 80°C 15 sec.	Zhao et al. (2019b)
AOA *amoA*	Arch-amoA F<br>Arch-amoA R	STAATGGTCTGGCTTAGACG<br>GCGGCCATCCATCTGTATGT	95°C 2 min, 40 cycles 95°C 15 sec, 55°C 60 sec, 80°C 15 sec.	Francis et al. (2005)
AOB *amoA*	amoA-1F<br>amoA-2R	GGGGTTTCTACTGGTGGT<br>CCCCTCKGSAAAGCCTTCTTC	95°C 2 min, 40 cycles 95°C 15 sec, 55°C 60 sec, 80°C 15 sec.	Rotthauwe et al. (1997)

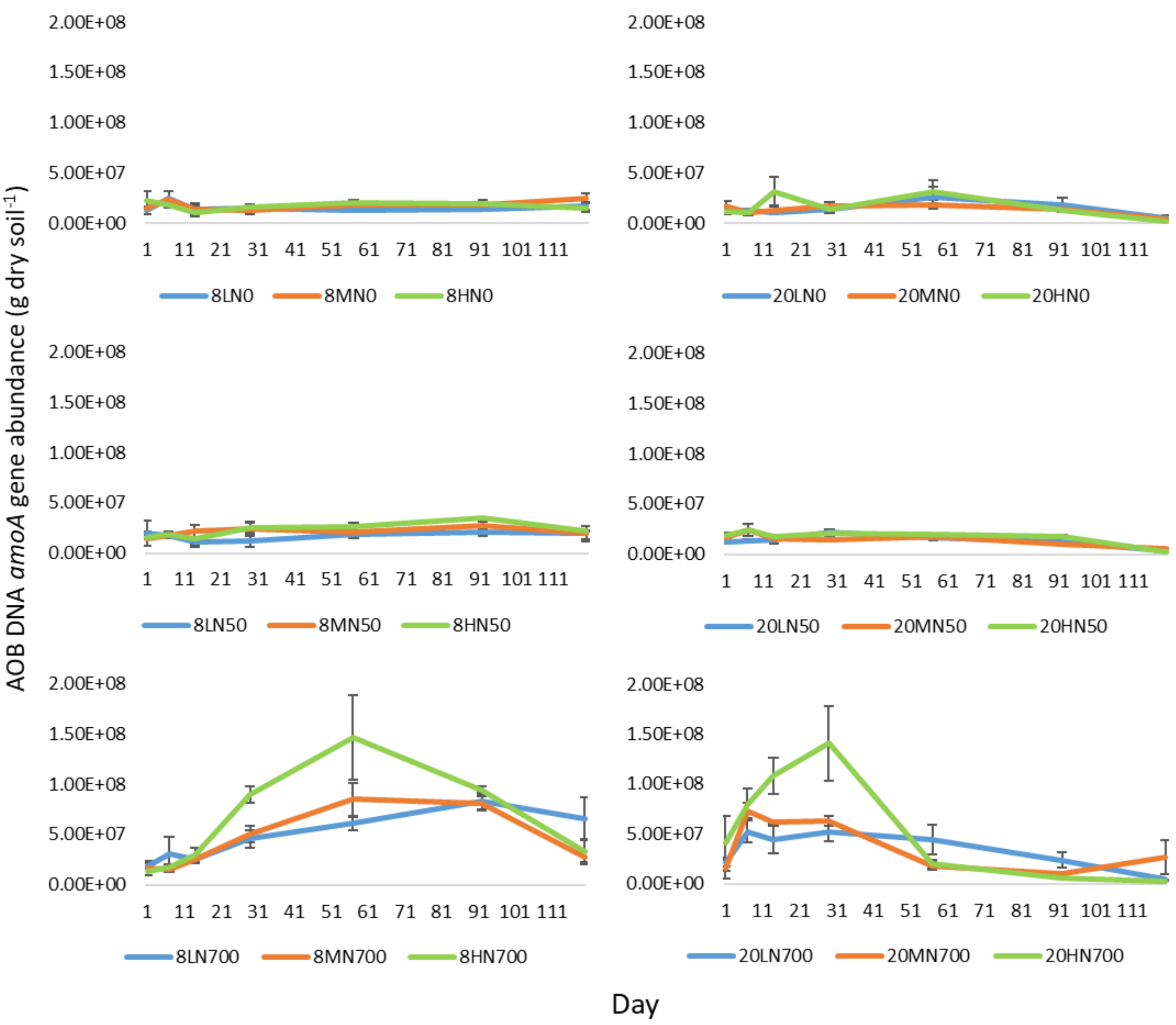

Supplementary Figure 1. AOB *amoA* DNA abundance. 8, 8 °C; 20, 20 °C; L, low moisture (28.5 % θg); M, medium moisture (37.5 % θg); H, high moisture (46.5 % θg); N0, no added nitrogen; N50, N applied at a rate of 50 kg N ha^{-1} as urea; N700, N applied at a rate of 700 kg N ha^{-1} as synthetic urine.

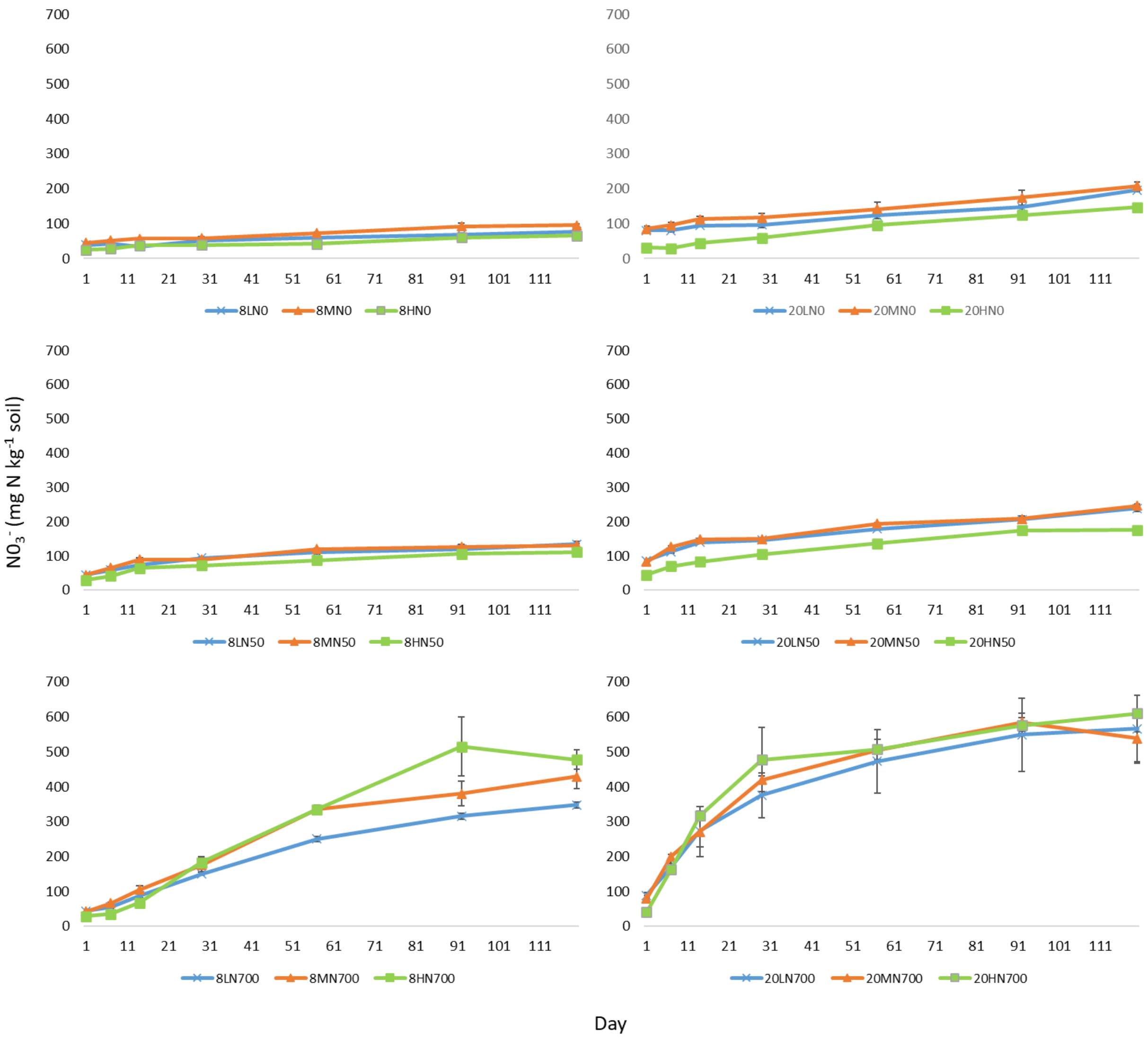

Supplementary Figure 2. Nitrate concentration within each treatment. 8, 8 °C; 20, 20 °C; L, low moisture (28.5 % θg); M, medium moisture (37.5 % θg); H, high moisture (46.5 % θg); N0, no added nitrogen; N50, N applied at a rate of 50 kg N ha^{-1} as urea; N700, N applied at a rate of 700 kg N ha^{-1} as synthetic urine.

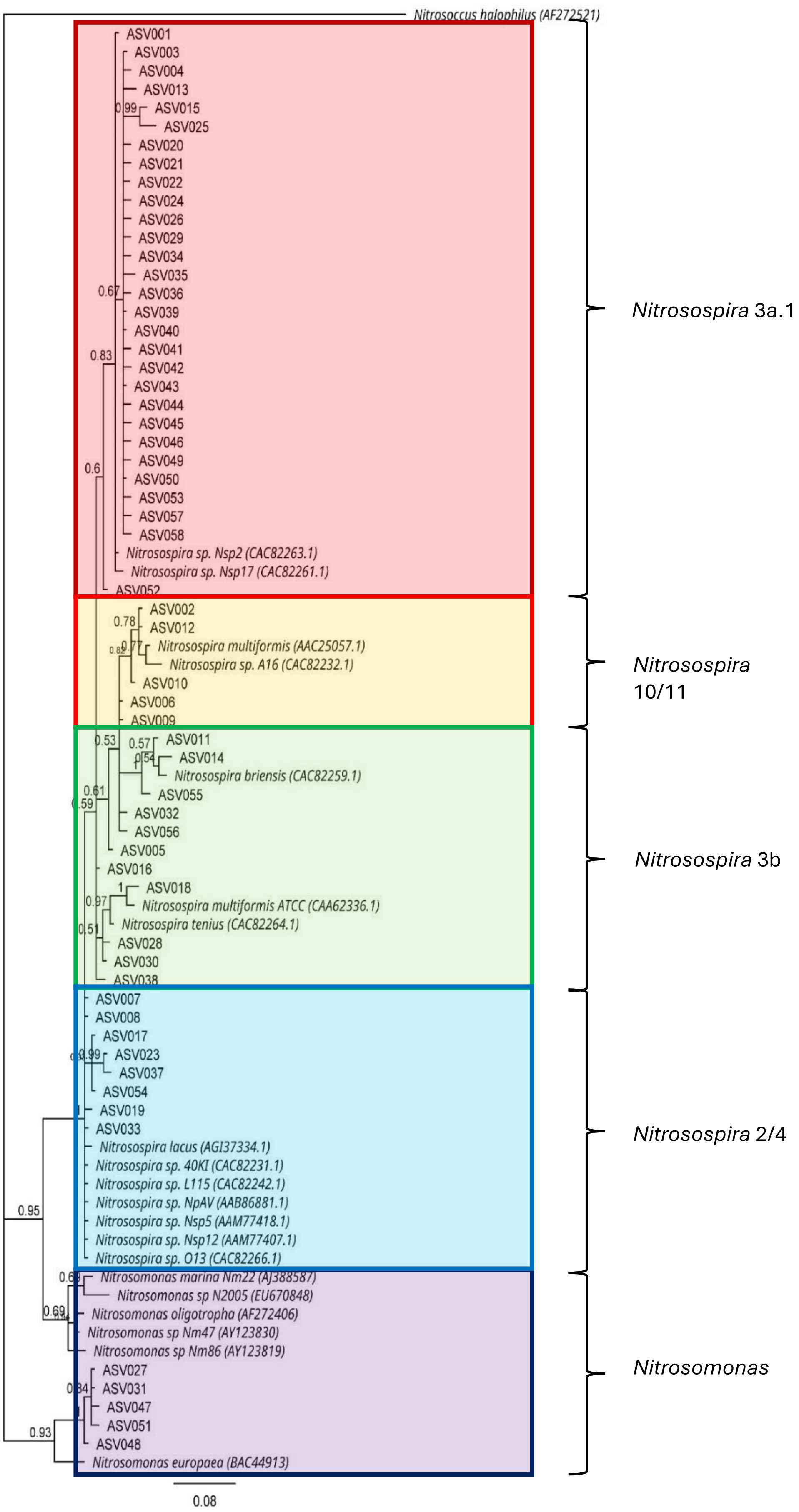

Nitrosoccus halophilus (AF272521)
ASV001
ASV003
ASV004
ASV013
0.99
ASV015
ASV025
ASV020
ASV021
ASV022
ASV024
ASV026
ASV029
ASV034
ASV035
0.67
ASV036
ASV039
ASV040
0.83
ASV041
ASV042
ASV043
ASV044
ASV045
ASV046
ASV049
0.6
ASV050
ASV053
ASV057
ASV058
Nitrosospira sp. Nsp2 (CAC82263.1)
Nitrosospira sp. Nsp17 (CAC82261.1)
ASV052
ASV002
0.78
ASV012
0.77
Nitrosospira multiformis (AAC25057.1)
Nitrosospira sp. A16 (CAC82232.1)
ASV010
ASV006
ASV009
0.53
0.57
ASV011
ASV014
Nitrosospira briensis (CAC82259.1)
ASV055
0.61
0.59
ASV032
ASV056
ASV005
ASV016
1
ASV018
0.97
Nitrosospira multiformis ATCC (CAA62336.1)
0.51
Nitrosospira tenius (CAC82264.1)
ASV028
ASV030
ASV038
ASV007
ASV008
ASV017
0.99
ASV023
ASV037
ASV054
ASV019
ASV033
Nitrosospira lacus (AGI37334.1)
Nitrosospira sp. 40KI (CAC82231.1)
Nitrosospira sp. L115 (CAC82242.1)
Nitrosospira sp. NpAV (AAB86881.1)
0.95
Nitrosospira sp. Nsp5 (AAM77418.1)
Nitrosospira sp. Nsp12 (AAM77407.1)
Nitrosospira sp. O13 (CAC82266.1)
0.69
Nitrosomonas marina Nm22 (AJ388587)
Nitrosomonas sp N2005 (EU670848)
0.69
Nitrosomonas oligotropha (AF272406)
Nitrosomonas sp Nm47 (AY123830)
Nitrosomonas sp Nm86 (AY123819)
ASV027
0.84
ASV031
ASV047
ASV051
0.93
ASV048
Nitrosomonas europaea (BAC44913)
0.08
Nitrosospira 3a.1
Nitrosospira 10/11
Nitrosospira 3b
Nitrosospira 2/4
Nitrosomonas

Supplementary Figure 3. Neighbour-joining tree of representative AOB *amoA* amino acid sequences. *Nitrosocuss halophilus* was used as an outgroup.

Supplementary Table 3. Spearman correlation analysis between the relative abundance of AOB clades and environmental parameters.

	Temperature	Moisture	pH	Ammonia	Ammonium	Nitrate
Nitrosospira 3a.1	- 0.46	-	-	-	-	- 0.43
Nitrosospira 10/11	0.32	0.33	-	-	-	-
Nitrosospira 3b	-	-	-	-	-	-
Nitrosospira 2/4	0.48	-	-	- 0.24	- 0.31	0.17
Nitrosomonas	0.47	-	-	0.33	-	-

Supplementary Table 4. Spearman correlation analysis between the relative abundance of AOA ASVs and environmental parameters.

	Temperature	Moisture	pH	Ammonia	Ammonium	Nitrate
ASV001	- 0.39	-	-	-	-	-
ASV002	- 0.74	-	0.38	0.25	0.40	- 0.29
ASV003	- 0.33	-	-	-	-	- 0.29
ASV004	- 0.55	-	0.28	-	0.25	- 0.25
ASV005	- 0.60	-	0.26	-	-	- 0.31
ASV006	- 0.76	-	0.44	-	0.35	- 0.33
ASV007	- 0.67	-	0.29	-	0.31	- 0.34
ASV009	0.61	-	- 0.49	-	- 0.43	-
ASV011	0.25	-	-	-	-	-
ASV015	- 0.40	-	-	-	-	- 0.24

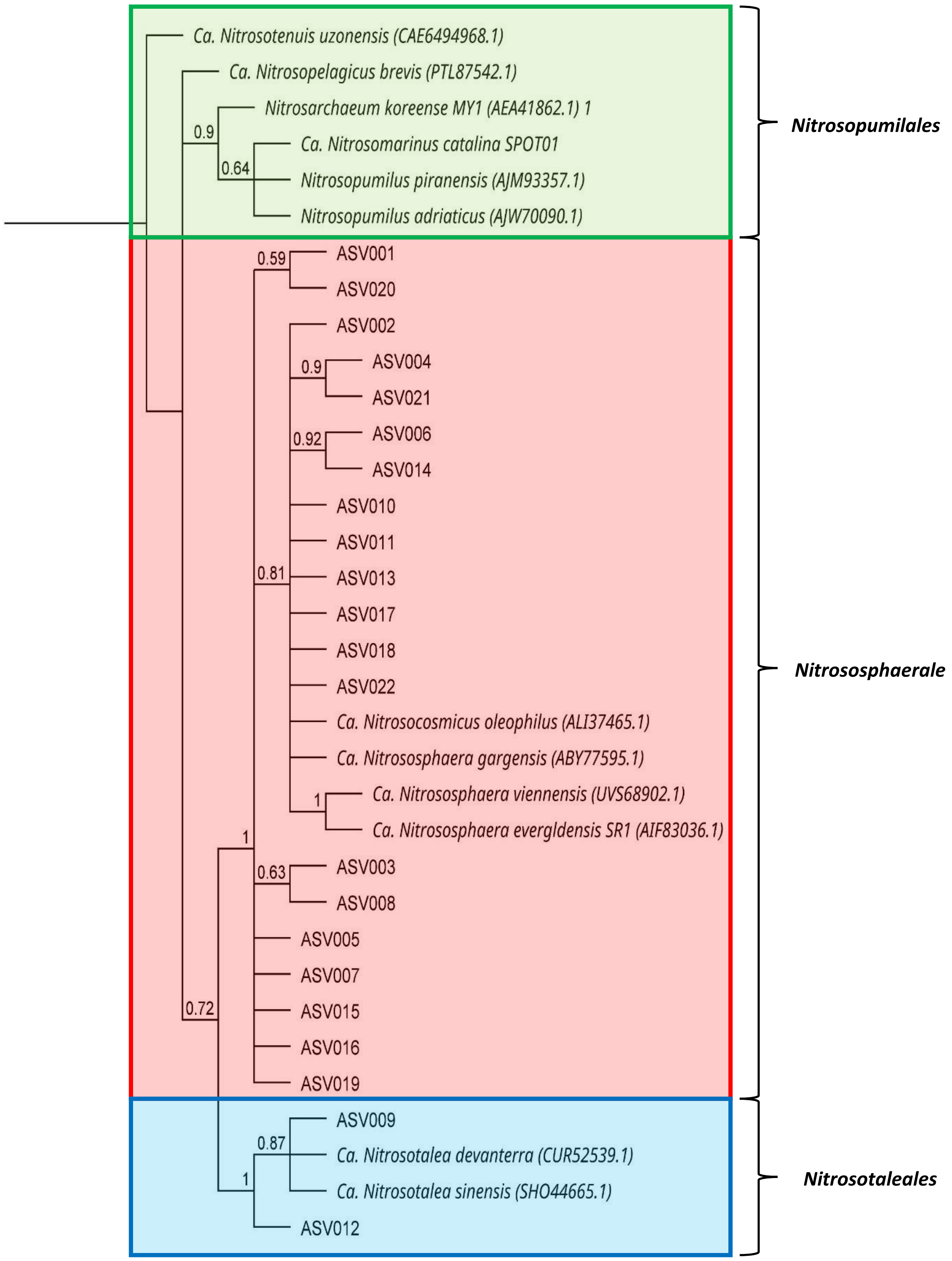

Ca. Nitrosotenuis uzonensis (CAE6494968.1)
Ca. Nitrosopelagicus brevis (PTL87542.1)
Nitrosarchaeum koreense MY1 (AEA41862.1) 1
Ca. Nitrosomarinus catalina SPOT01
Nitrosopumilus piranensis (AJM93357.1)
Nitrosopumilus adriaticus (AJW70090.1)
0.9
0.64
Nitrosopumilales
ASV001
ASV020
0.59
ASV002
ASV004
ASV021
0.9
ASV006
ASV014
0.92
ASV010
ASV011
ASV013
0.81
ASV017
ASV018
ASV022
Ca. Nitrosocosmicus oleophilus (ALI37465.1)
Ca. Nitrososphaera gargensis (ABY77595.1)
Ca. Nitrososphaera viennensis (UVS68902.1)
Ca. Nitrososphaera evergldensis SR1 (AIF83036.1)
1
ASV003
ASV008
0.63
ASV005
ASV007
ASV015
ASV016
ASV019
0.72
Nitrososphaerale
ASV009
Ca. Nitrosotalea devanterra (CUR52539.1)
Ca. Nitrosotalea sinensis (SHO44665.1)
0.87
1
ASV012
Nitrosotaleales

Supplementary Figure 4. Neighbour-joining tree of representative AOA *amoA* amino acid sequences.

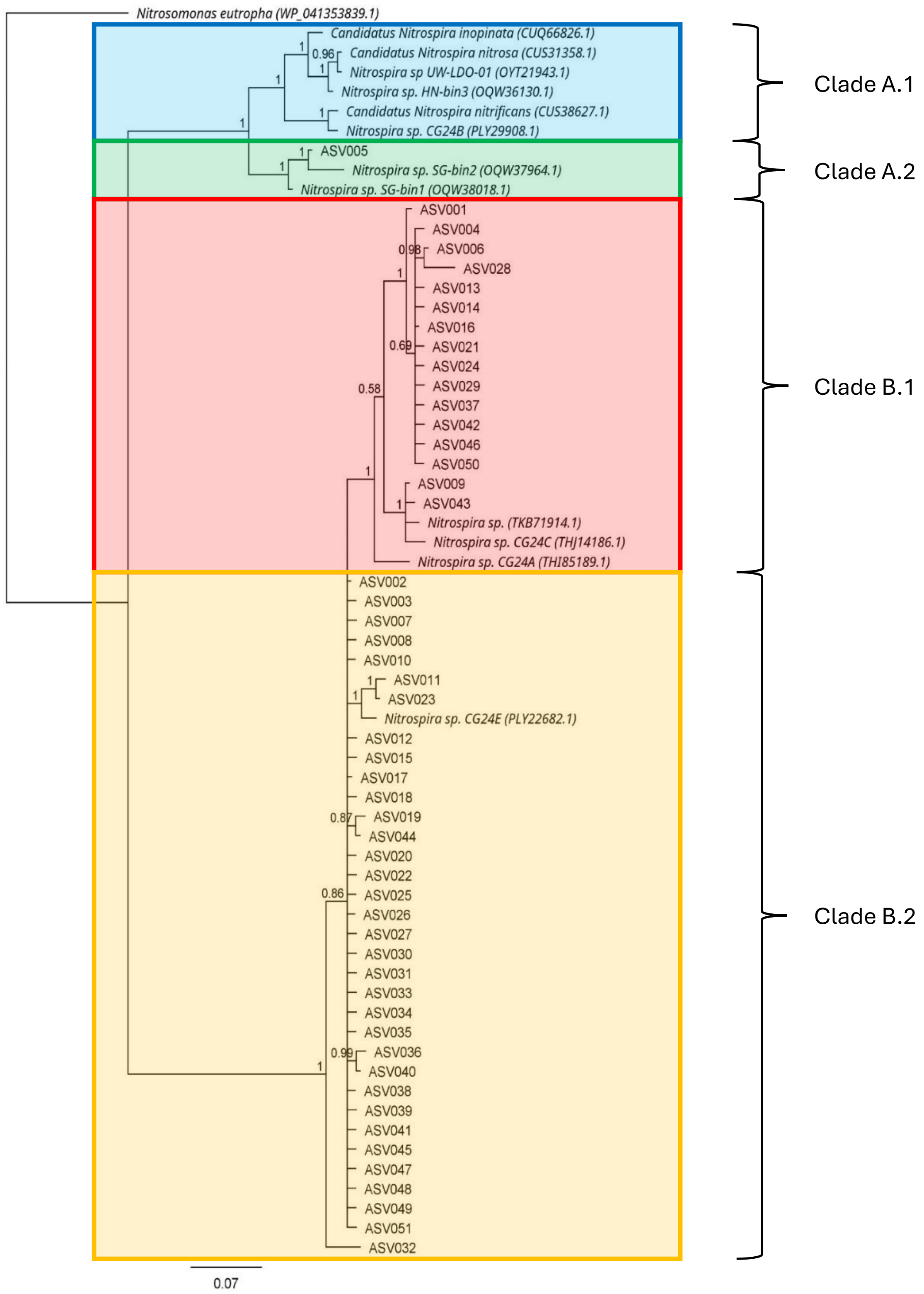

Supplementary Figure 5. Neighbour-joining tree of representative comammox *amoA* amino acid sequences. *Nitrosomonas eutropha* is used as an outgroup.

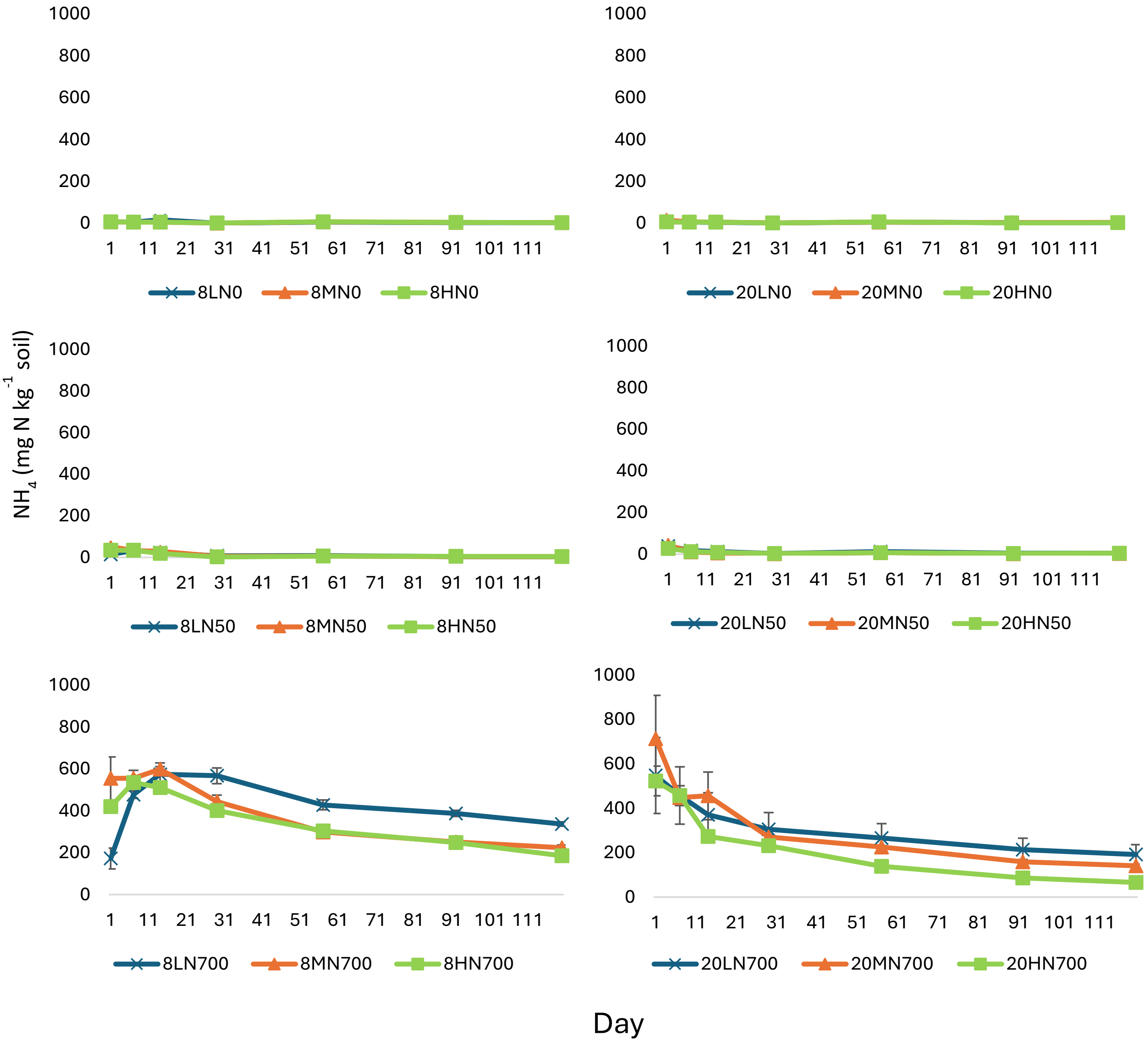

Supplementary Figure 6. Soil ammonium concentration within each treatment. 8, 8 °C; 20, 20 °C; L, low moisture (28.5 % θg); M, medium moisture (37.5 % θg); H, high moisture (46.5 % θg); N0, no added nitrogen; N50, N applied at a rate of 50 kg N ha^{-1} as urea; N700, N applied at a rate of 700 kg N ha^{-1} as synthetic urine.

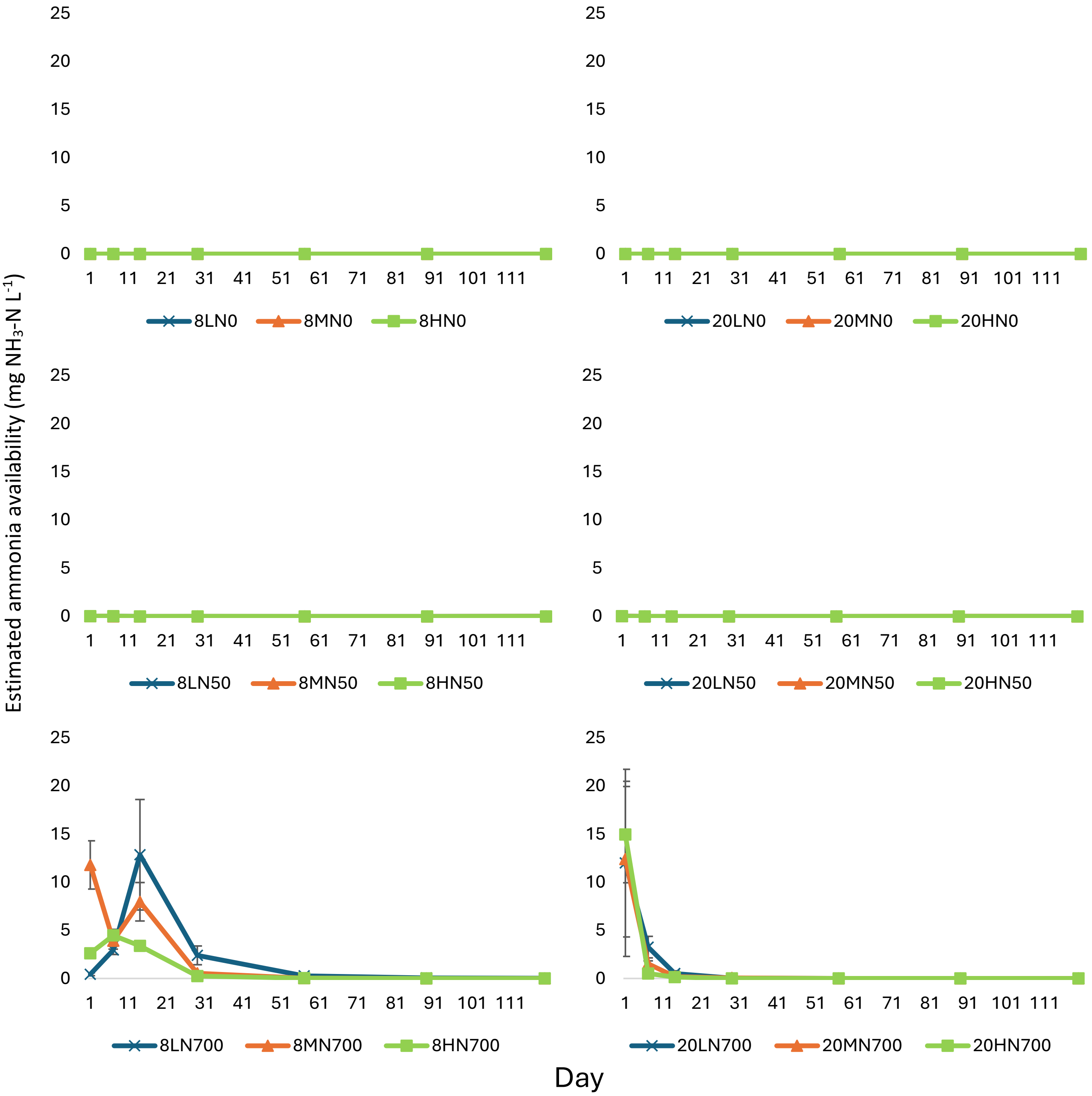

Supplementary Figure 7. Estimated soil ammonia availability within each treatment. 8, 8 °C; 20, 20 °C; L, low moisture (28.5 % θg); M, medium moisture (37.5 % θg); H, high moisture (46.5 % θg); N0, no added nitrogen; N50, N applied at a rate of 50 kg N ha^{-1} as urea; N700, N applied at a rate of 700 kg N ha^{-1} as synthetic urine

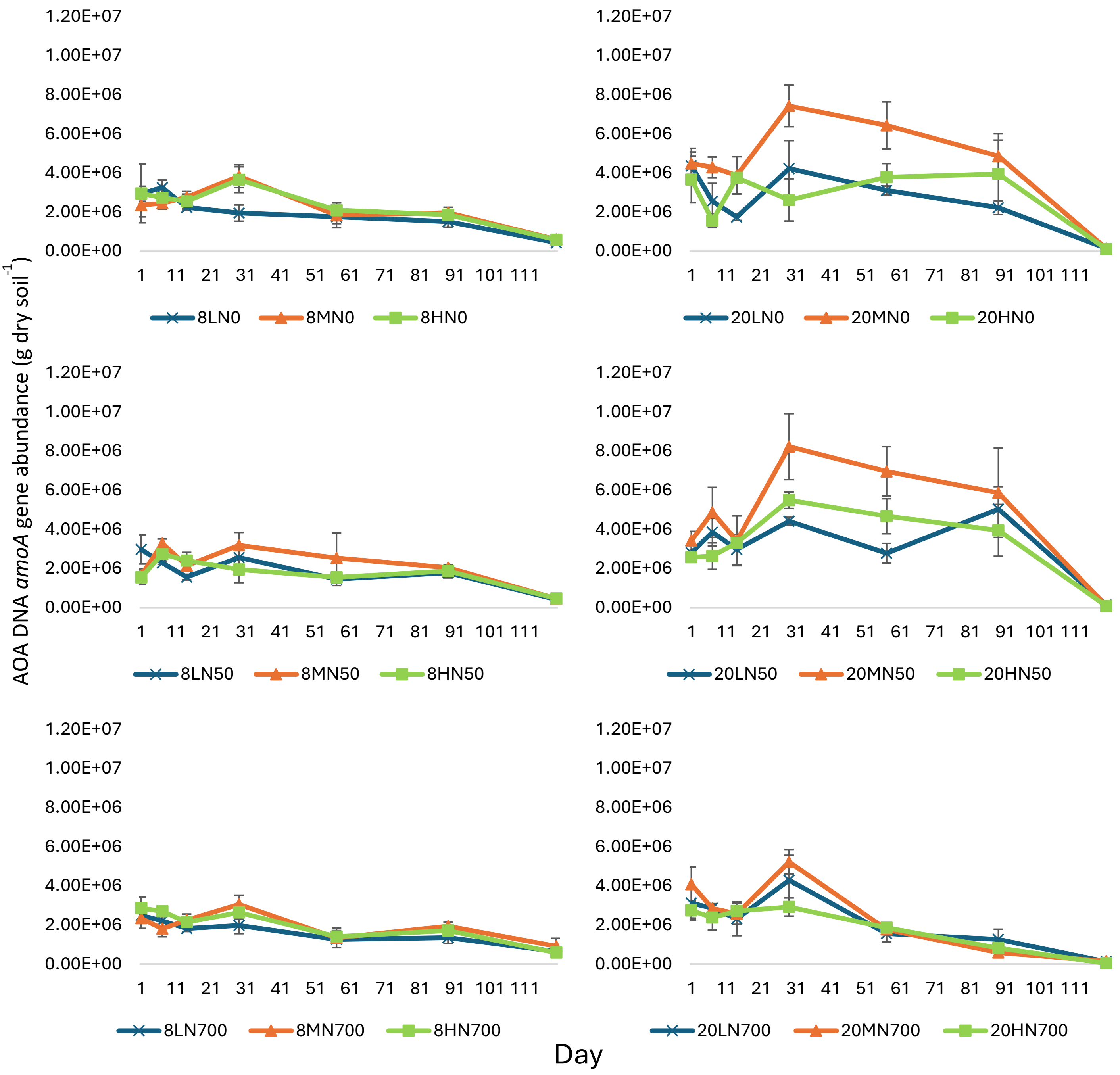

Supplementary Figure 8. AOA *amoA* DNA abundance. 8, 8 °C; 20, 20 °C; L, low moisture (28.5 % θg); M, medium moisture (37.5 % θg); H, high moisture (46.5 % θg); N0, no added nitrogen; N50, N applied at a rate of 50 kg N ha^{-1} as urea; N700, N applied at a rate of 700 kg N ha^{-1} as synthetic urine.

Supplementary Table 5: Soil pH from the treatments at each time point. 8, 8 °C. 20, 20 °C. L, low moisture (28.5% gravimetric water content). M, medium moisture (37.5% gravimetric water content). H, high moisture (46.5% gravimetric water content). N0, no nitrogen. N50, 50 kg urea-N ha^{-1}. N700, 700 kg synthetic urine-N ha^{-1}.

Treatment	Day 1	Day 7	Day 14	Day 29	Day 57	Day 92	Day 120
8LN0	5.5	5.5	5.5	5.5	5.4	5.3	5.3
8LN50	5.5	5.5	5.4	5.3	5.2	5.0	5.0
8LN700	6.6	7.2	7.6	6.8	6.2	5.6	5.3
8MN0	5.4	5.4	5.4	5.5	5.3	5.2	5.1
8MN50	5.8	5.4	5.4	5.2	5.1	5.0	5.0
8MN700	7.9	7.3	7.6	6.5	5.4	5.0	4.8
8HN0	5.7	5.7	5.7	5.6	5.5	5.4	5.3
8HN50	5.7	5.7	5.4	5.3	5.3	5.1	5.1
8HN700	7.4	7.5	7.4	6.3	5.5	5.0	4.6
20LN0	5.3	5.3	5.3	5.2	5.1	4.9	4.8
20LN50	5.3	5.2	5.1	5.0	4.9	4.7	4.7
20LN700	7.2	6.8	6.1	5.2	4.9	4.5	4.4
20MN0	5.4	5.3	5.2	5.1	5.0	4.9	4.8
20MN50	5.3	5.1	5.1	5.0	4.9	4.8	4.7
20MN700	7.2	6.6	5.7	5.2	4.7	4.4	4.3
20HN0	5.7	5.7	5.6	5.4	5.3	5.1	5.1
20HN50	5.6	5.5	5.4	5.3	5.0	5.0	4.9
20HN700	7.6	6.2	5.5	5.0	4.7	4.3	4.3

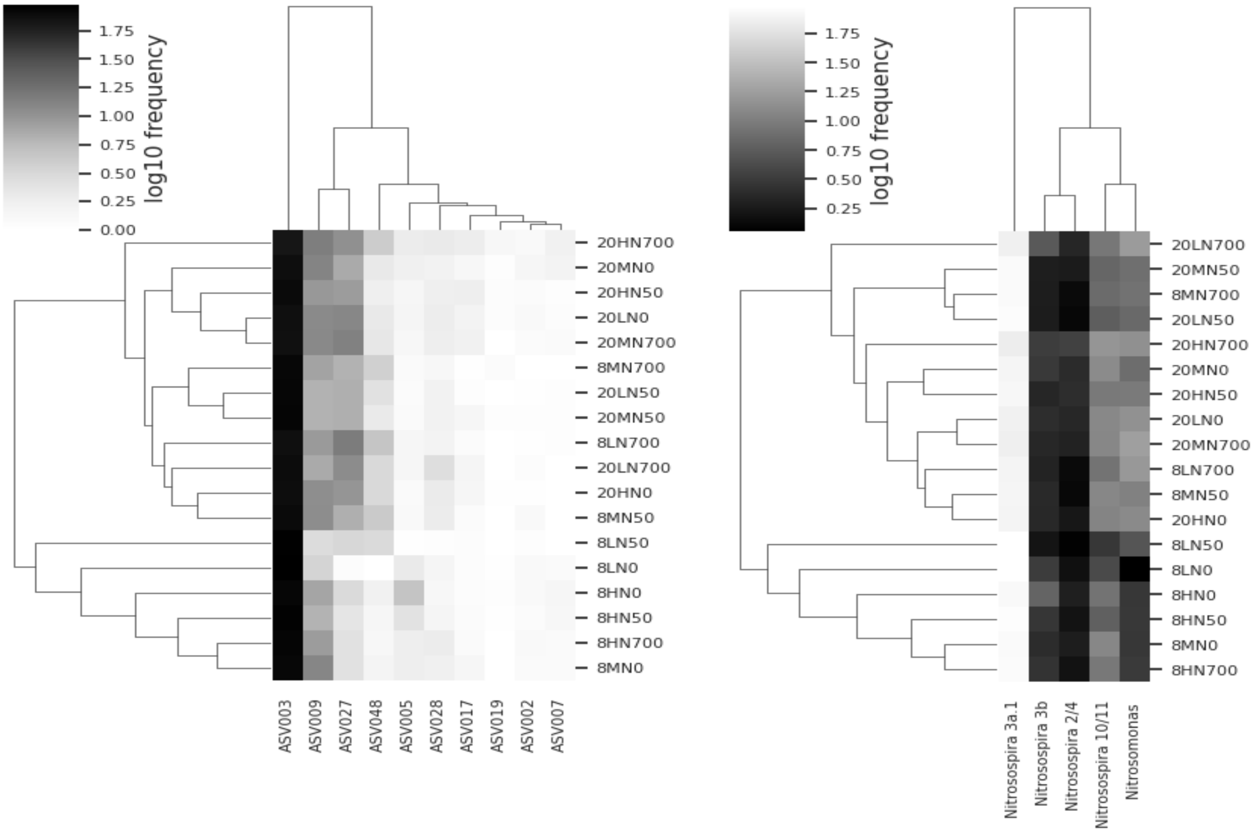

Supplementary Figure 9. Heatmap of the common AOB ASVs (left) and the AOB clades within each treatment. Relative abundance of clades is based on the associated ASVs shown in the phylogenetic tree (Supplementary Figure 3). 8, 8 °C; 20, 20 °C; L, low moisture (28.5 % θg); M, medium moisture (37.5 % θg); H, high moisture (46.5 % θg); N0, no added nitrogen; N50, N applied at a rate of 50 kg N ha^{-1} as urea; N700, N applied at a rate of 700 kg N ha^{-1} as synthetic urine.

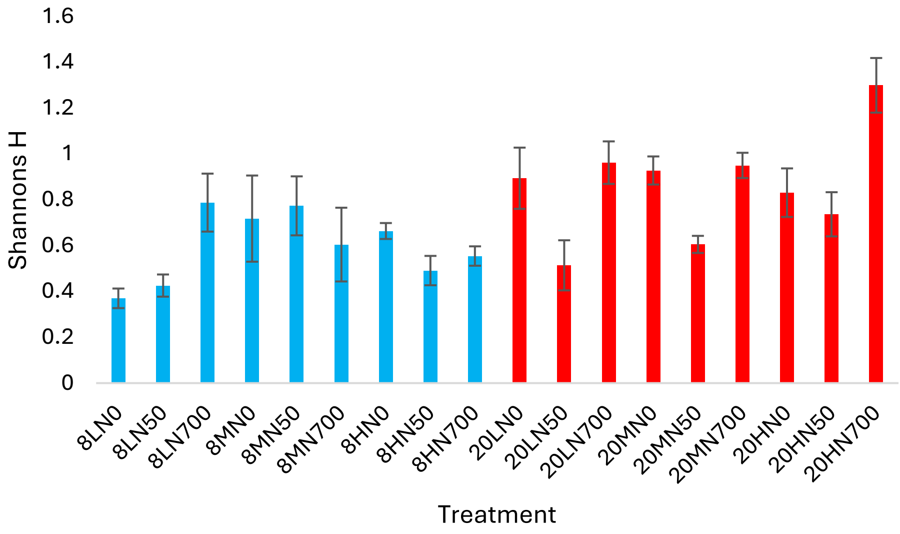

Supplementary Figure 10. Shannons entropy of the AOB community in each treatment. Error bars represent standard error of the mean. 8, 8 °C; 20, 20 °C; L, low moisture (28.5 % θg); M, medium moisture (37.5 % θg); H, high moisture (46.5 % θg); N0, no added nitrogen; N50, N applied at a rate of 50 kg N ha-1 as urea; N700, N applied at a rate of 700 kg N ha-1 as synthetic urine.

Supplementary Table 6. Spearman correlation of Shannons H entropy for AOA, AOB, and comammox *Nitrospira* (COM) and environmental parameters at day 14. Dashs (-) indicate the correlation was non-significant ($P > 0.05$)

	Temperature	pH	NH_4-N	NH_3-N	NO_3^--N
AOB	0.49	-	-	-	0.48
AOA	- 0.63	-	0.21	-	- 0.34
COM	- 0.29	0.52	-	0.25	- 0.40

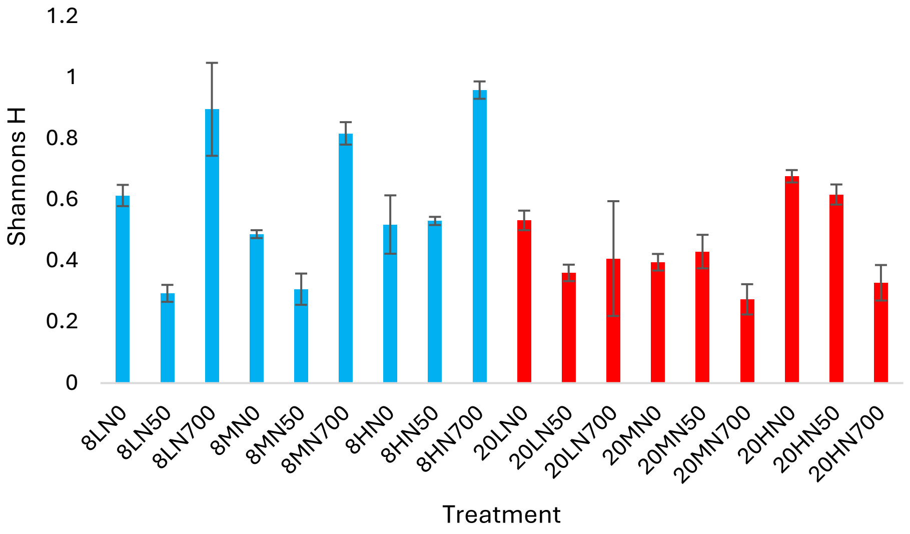

Supplementary Figure 11. Shannons entropy of the comammox *Nitrospira* community in each treatment. 8, 8 °C; 20, 20 °C; L, low moisture (28.5 % θg); M, medium moisture (37.5 % θg); H, high moisture (46.5 % θg); N0, no added nitrogen; N50, N applied at a rate of 50 kg N ha^{-1} as urea; N700, N applied at a rate of 700 kg N ha^{-1} as synthetic urine.

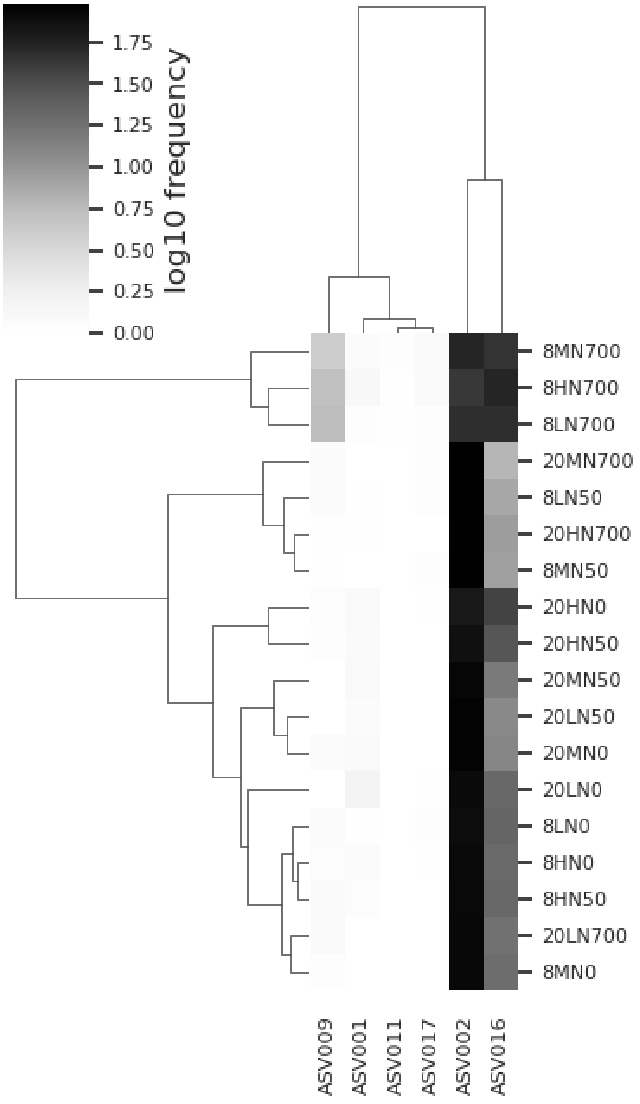

Supplementary Figure 12. Heatmap of the common comammox *Nitrospira* ASVs. 8, 8 °C; 20, 20 °C; L, low moisture (28.5 % θg); M, medium moisture (37.5 % θg); H, high moisture (46.5 % θg); N0, no added nitrogen; N50, N applied at a rate of 50 kg N ha^{-1} as urea; N700, N applied at a rate of 700 kg N ha^{-1} as synthetic urine.

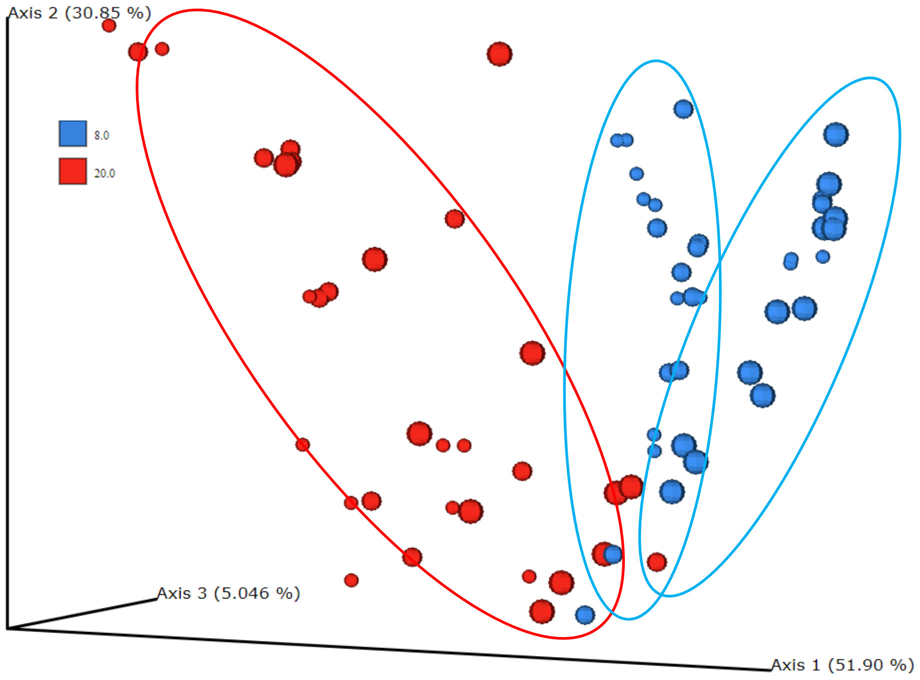

Supplementary Figure 13. Bray-Curtis analysis of the AOA community. Red represents AOA communities from the 20 °C samples. Blue represents AOA communities from the 8 °C samples. Size of the points represents the amount of nitrogen added.

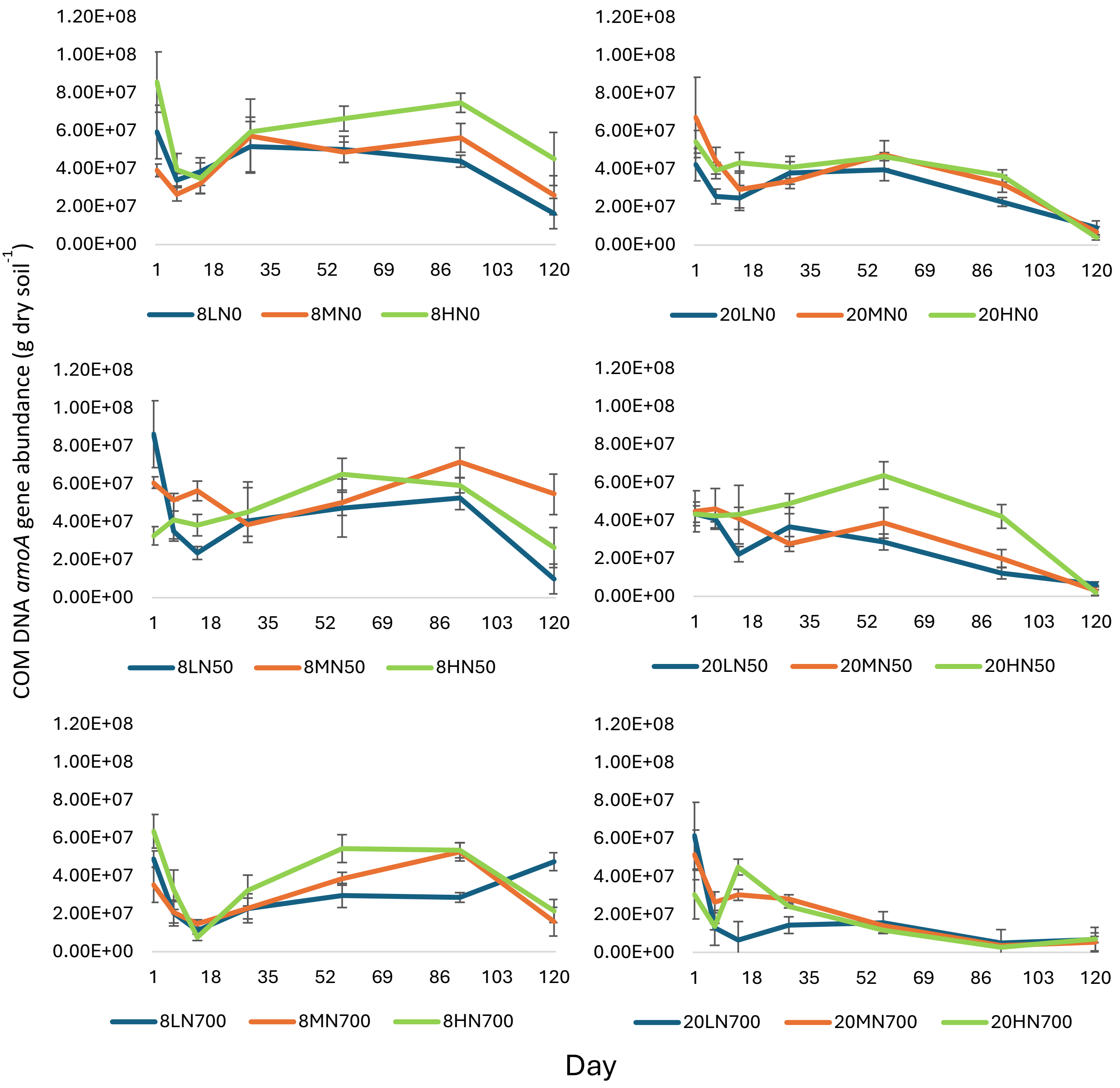

Supplementary Figure 14. COM *amoA* DNA abundance. 8, 8 °C; 20, 20 °C; L, low moisture (28.5 % θg); M, medium moisture (37.5 % θg); H, high moisture (46.5 % θg); N0, no added nitrogen; N50, N applied at a rate of 50 kg N ha^{-1} as urea; N700, N applied at a rate of 700 kg N ha^{-1} as synthetic urine.
